# HyCas9-12aGEP: an efficient genome editing platform for *Corynebacterium glutamicum*

**DOI:** 10.3389/fbioe.2024.1327172

**Published:** 2024-03-12

**Authors:** Feng Zhang, Jin-Yu Wang, Chang-Lon Li, Wei-Guo Zhang

**Affiliations:** The Key Laboratory of Industrial Biotechnology, Ministry of Education, School of Biotechnology, Jiangnan University, Wuxi, China

**Keywords:** active gRNA, editing resolution, hfgRNA, editing efficiency, *Corynebacterium glutamicum*

## Abstract

*Corynebacterium glutamicum* plays a crucial role as a significant industrial producer of metabolites. Despite the successful development of CRISPR-Cas9 and CRISPR-Cas12a-assisted genome editing technologies in *C. glutamicum*, their editing resolution and efficiency are hampered by the diverse on-target activities of guide RNAs (gRNAs). To address this problem, a hybrid CRISPR-Cas9-Cas12a genome editing platform (HyCas9-12aGEP) was developed in *C*. *glutamicum* in this study to co-express sgRNA (corresponding to *Sp*Cas9 guide RNA), crRNA (corresponding to *Fn*Cas12a guide RNA), or hfgRNA (formed by the fusion of sgRNA and crRNA). HyCas9-12aGEP improves the efficiency of mapping active gRNAs and outperforms both CRISPR-Cas9 and CRISPR-Cas12a in genome editing resolution and efficiency. In the experiment involving the deletion of the *cg0697-0740* gene segment, an unexpected phenotype was observed, and HyCas9-12aGEP efficiently identified the responsible genotype from more than 40 genes. Here, HyCas9-12aGEP greatly improve our capability in terms of genome reprogramming in *C. glutamicum.*

## Introduction

The enzyme associated with clustered regularly interspaced short palindromic repeats (CRISPR), such as Cas9 or Cas12a, is an RNA-guided endonuclease that utilizes RNA–DNA base-pairing to identify and target foreign DNA within bacteria (Jinek et al., 2012; Zetsche et al., 2015). Guide RNA complexes with Cas9 or Cas12a are potent genome-engineering agents in both eukaryotes and prokaryotes, extensively employed in CRISPR-based methodologies (Cong et al., 2013; Jiang et al., 2013; Yan et al., 2017; Zetsche et al., 2017). For example, genetic engineering has been developed for *C. glutamicum* (Jiang et al., 2017; Liu et al., 2017; Peng et al., 2017; Wang et al., 2021; Liu et al., 2022). *C. glutamicum,* strategically engineered for industrial amino acid synthesis, serves as a versatile microorganism capable of producing a diverse range of compounds, including sunscreens, anti-aging sugars, biofuels, and polymers designed for regenerative medicine applications (Becker et al., 2018; Wolf et al., 2021). Despite being regarded as a promising tool for genome engineering *in C. glutamicum*, CRISPR-Cas9 or CRISPR-Cas12a encounters challenges primarily due to the varied on-target activities of guide RNAs (gRNAs), posing potential obstacles to its successful development.

Cas9 and Cas12a recognize distinct protospacer adjacent motif (PAM) sequences, for instance, *Sp*Cas9 and *Fn*Cas12a specifically recognize 5′-NGG-3′ and 5′-TTN-3′, respectively (Jinek et al., 2012; Zetsche et al., 2015). A major challenge in CRISPR/Cas9-and Cas12a-mediated genome engineering is that not all guide RNAs (gRNAs) efficiently cleave the DNA (Doench et al., 2014; Jiang et al., 2017; Kim et al., 2018; Creutzburg et al., 2020; Zhang et al., 2020; Corsi et al., 2022). Therefore, the selection and design of active gRNA are critical for CRISPR-Cas9-mediated gene editing. However, the efficacy of guide RNA (gRNA) is impacted by various factors, encompassing gRNA structure (Abdel-Mawgoud and Stephanopoulos, 2020; Creutzburg et al., 2020; Magnusson et al., 2021; Riesenberg et al., 2022), conformational transitions (Chen et al., 2017; Wang et al., 2019; Kim et al., 2020; Talas et al., 2021), R-loop formation (Gong et al., 2018), Cas9 and Cas12a variants (Chen et al., 2017; Guo et al., 2018; Wang et al., 2019; Kim et al., 2020), PAM sequences (Doench et al., 2014), supercoiling (Ivanov et al., 2020), DNA covalent modification (Tao et al., 2017; Vlot et al., 2018; Liu Y et al., 2020; Dong et al., 2021), interactions at non-targeted sites (Sternberg et al., 2014; Moreb et al., 2020), target copy number (Ivanov et al., 2020), and target accessibility (Chen et al., 2016; Horlbeck et al., 2016; Yarrington et al., 2018). Despite extensive screening of large guide RNA (gRNA) libraries and the development of algorithms to predict sequence-dependent gRNA activity (Park et al., 2021; Talas et al., 2021; Xiang et al., 2021), these algorithms exhibit limitations in accurately predicting other datasets, training datasets, or variations across different species (Moreb and Lynch, 2021). Thus, beyond the utilization of current gRNA design tools, the central challenge in gene editing resides in the experimental strategies to promptly identify active gRNAs and enhance their efficacy.

Off-target effects pose a critical challenge in CRISPR-Cas9-based gene editing and disease therapy. These effects are influenced by various factors, encompassing the quality of guide gRNA, the selection of the Cas9 protein, concentrations of both gRNA and Cas protein, cell type, and the choice of target site (Pattanayak et al., 2013; Wienert and Cromer, 2022; Guo et al., 2023). Among these factors, gRNA concentration and the level of Cas9 protein expression are the primary contributors (Hsu et al., 2013; Pattanayak et al., 2013). The effectiveness of the CRISPR-Cas9 system depends on sufficient Cas9 protein expression, as Cas9 serves as the actual nuclease responsible for gene editing. Insufficient Cas9 protein expression can impair its ability to accurately identify and cleave the target DNA, reducing editing efficiency (Pattanayak et al., 2013). Conversely, an excessive Cas9 protein concentration may lead to cleavage at off-target sites with partial sequence similarity, thereby increasing the risk of off-target effects (Hsu et al., 2013). Thus, in CRISPR-Cas9 experiments, it is essential to maintain a balanced Cas9 protein expression level to ensure efficient editing while minimizing the potential for off-target effects.

In this study, we present HyCas9-12aGEP, a system that involves the co-expression of *Streptococcus pyogenes* (*Sp*)-Cas9 and *Francisella novicida* (*Fn*)-Cas12a nucleases by integrating the *Sp*cas9 and *Fn*cas12a genes into *C. glutamicum*. This system utilizes 'hybrid fused guide’ (hfg)RNAs, generated by fusing *Sp*Cas9 and *Fn*Cas12a guide RNAs and expressed from a single promoter. By reducing the expression level of *Sp*Cas9, HyCas9-12aGEP mitigates the off-target effects associated with *Sp*Cas9. In comparison to conventional CRISPR-Cas9 or Cas12a systems, HyCas9-12aGEP, coupled with hfgRNA, markedly augments guide RNA (gRNA) activity, streamlines the identification of active gRNAs, and enhances the efficiency of gene editing. Additionally, *Sp*Cas9 and *Fn*Cas12a recognize different PAM sequences, specifically 5′-NGG-3′ and 5′-TTN-3′, respectively. Therefore, the active gRNAs (corresponding to an active PAM) that HyCas9-12aGEP can use are the sum of *Sp*Cas9 and *Fn*Cas12a, which improves the resolution of gene editing, such as precise substitution of the 149th Glycine with Lysine in the γ-glutamyl kinase encoded by *proB*. Interestingly, our experiments unveiled a notable phenotype in colonies with a 40.96 kb DNA segment (*cg0697*-*cg0740*) deleted from the *C. glutamicum* genome, exhibiting increased “moisture.” Using HyCas9-12aGEP, genotypes that influence the phenotype were rapidly mapped from a pool of over 40 genes. In summary, HyCas9-12aGEP emerges as a potent tool for genetically modifying *C. glutamicum*, accelerating research in gene function, and optimizing the production of target products.

## Materials and methods

### Strains and culture conditions

Strains used in this study are listed in Supplementary Table S1. *E. coli JM109*, utilized for plasmid cloning, was aerobically cultivated at 37°C in Luria–Bertani (LB) broth. The medium was supplemented accordingly with kanamycin (Kan, 50 μg/mL) or chloramphenicol (Cm, 20 μg/mL). *C. glutamicum* were cultured at 30°C in LBG medium (LB medium supplemented with 5 g/L glucose). The Epo medium, consisting of LBG supplemented with 3% glycine, 0.1% Tween 80, and 0.4% isoniazid, was utilized for cultivating electroporation-competent cells. LBHIS medium, containing 2.5 g/L yeast extract, 5 g/L tryptone, 5 g/L NaCl, 18.5 g/L Brain Heart Infusion powder, and 91 g/L sorbitol, were employed to obtain transformants of *C. glutamicum*, following previously described procedures (Xu J et al., 2014). The CM medium (10 g/L yeast extract, 10 g/L beef extract, 10 g/L tryptone, 5 g/L glucose, and 5 g/L NaCl) is utilized for colony phenotype observation on agar plates. Kan (25 μg/mL) was added to LBHIS medium as required.

### Plasmid construction

Plasmids utilized in this study are listed in Supplementary Table S2 and Supplementary Data S1. Plasmids were constructed via recombination or T4 DNA ligase. Recombination was conducted using the ClonExpress II and ClonExpress MultiS One Step Cloning Kit (Vazyme, Nanjing, China). Restriction endonucleases and T4 DNA ligase were purchased from TaKaRa (Dalian, China). DNA polymerase and reagents were purchased from Vazyme (Nanjing, China). Gene synthesis and DNA sequencing were provided by GENEWIZ Inc. (Suzhou, China). Primers synthesized by Exsyn-bio (Wuxi, China) and details for constructing plasmids are described in Supplementary Data S1.

### Design of gRNAs

The general sgRNAs were designed via online software (http://www.rgenome.net/cas-designer/) (Park J and Kim, 2015). The relevant sgRNA sequences are listed in Supplementary Data S2.

### Genome editing in *C. glutamicum*

The traditional pK18*mobsacB*–based gene deletion and insertion were performed as previously described (Xu J et al., 2014). HyCas9-12aGEP has been successfully applied in genome editing, and the detail progress are listed in the Supplementary methods (Supplementary Material). In this study, the size of homologous arm (HA) carried by plasmid pZF2 was −1 kb.

The preparation of competent *C. glutamicum* was carried out following the previously described method with appropriate modifications (Liu et al., 2022). The strains were cultured in 50-mL shake flasks with 10 mL of LBG media for 10–13 h, and then 3 mL was transferred to 100 mL of Epo media in 500-mL shake flasks for 30°C-cultivation. When the **△**OD_600_ of the culture reached to 0.4-0.5, the culture were ice-bathed for 15–20 min and were then harvested through 5-min centrifugation at 4°C and 4,000 rpm. The cells were subsequently resuspended in 300–500 μL of 10.0% (v/v) glycerol after washing 3 times using 4°C pre-chilled 10% glycerol. The plasmid was mixed with competent cells and subsequently introduced into an electroporation cuvette. Electroporation was carried out utilizing an GenePulser Xcell™ (Bio-Rad Laboratories, Shanghai, China) with parameter settings of 1800 V, 5 ms, and 1 mm. Subsequently, 800 μL of LBHIS media was immediately added, followed by rapidly 6-min incubation of the suspension at 46°C. The cells were 2-h cultured at 30°C, and then spread on LBHIS plates containing antibiotics for 30°C-incubation until the apperance of colonies.

### Re-sequencing analysis

Re-sequencing was conducted to identify off-target occurrences in the edited strains. Referring to the previously described method (Peng et al., 2017). Total DNA from C. glutamicum was extracted following the manufacturer’s protocol provided by Vazyme, Nanjing, China. The assessment of DNA quality involved utilizing the Qubit Fluorometer (Thermo Fisher Scientific, San Jose, CA, United States) for measuring overall mass and the Fragment Analyzer for evaluating DNA integrity. The genomic sequencing was executed utilizing the Illumina HiSeq/Nova 2 × 150bp system (Illumina, San Diego, CA, United States) at the GENEWIZ Inc. (Suzhou, China).

### Plasmid curing

For plasmid curing, transformants were grown in antibiotic-free LBG medium at 37°C overnight (−8 h) and subsequently plated on antibiotic-free CM plates. The next day, the culture is diluted and coated into kanamycin-resistant CM plates and cultured at 30°C. Plasmid curing was judged by the colony’s sensitivity to antibiotics. The grown single colonies were transferred to one CM kanamycin-resistant plate and the corresponding CM plate, respectively. Single colonies that cannot be grown in response to resistant plates are plasmid eliminated successfully for the next round of gene editing or other tests.

### RT-PCR for mRNA quantification

RT-qPCR assay was performed as described previously (Wang et al., 2020). To determine the transcriptional intensity of *Spcas9* or *Fncas12a* in the seven *Spcas9* or *Fncas12a*-expressing strains, i.e. 9-12, E9-12, L9-12, 9-E12, 9-L12, Cas9-2, and Cas12-2, quantitative reverse transcription PCR (qRT-PCR) was performed using total RNA samples. Total RNA was isolated from the cells with the RNAprep Pure Cell/Bacteria Kit (Tiangen, China). 0.5 μg of the total bacterial RNA was subjected to reverse transcription using the HiScript II Q RT SuperMix Kit (Vazyme). RT-qPCR analysis using the ChamQ Universal SYBR qPCR master mix kit (Vazyme) in a total reaction volume of 20 μL in a CFX96™ Real-Time System (Bio-Rad, Hercules, CA, United States). The 16S rRNA encoding gene was used as an internal control as described previously (Pan et al., 2022). PCR primers used in RT-PCR are listed in Supplementary Data S1.

### Analytical methods

Cell growth was calculated by measuring the optical density at 600 nm (OD_600_) using a spectrophotometer (Shanghai, China). Cell morphology was examined using field emission scanning electron microscopy (FESEM). C. glutamicum cells were harvested by centrifugation, washed thrice with physiological saline (pH 7.0), and subsequently deposited onto a small silicon platelet. After air-drying at room temperature, the cells underwent *in-situ* fixation with a 2.5% glutaraldehyde solution in a 0.15 M sodium phosphate buffer (pH 7.4) for 10 min. The samples were gold-coated and examined under field emission scanning electron microscopy (FESEM) using a Hitachi SU8220 instrument with an accelerating voltage of 3 kV.

### Statistical analysis

All experiments were conducted with three independent replicates. Statistical analysis of the data was carried out using t-tests in SPSS v.25. A significance threshold of *p* < 0.05 was applied, and the level of significance is denoted as ***p < 0.01.

## Results

### Exploration of gene editing system to broaden the target range

The success of CRISPR gene editing applications relies significantly on the presence of active gRNAs. *Sp*RY-Cas9 (Walton RT et al., 2020) is an unconstrained near-PAMless genome, and *Sp*Cas9-HF1 (Chen et al., 2017) is high-fidelity *Sp*Cas9 variant. Thus, the mutant *Sp*RY-HF1 obtained by combining *Sp*RY-Cas9 and *Sp*Cas9-HF1 was inferred to increase the density of active gRNAs and to improve editing resolution. Thus, the open reading frame (ORF) of *Sp*Cas9 in pFSC (Peng et al., 2017) was replaced with the codon-optimized ORF of SpRY-HF1 (NCBI accession numbers: OP345224) to obtain the plasmid pXMJ19-SpRY-HF1. Subsequently, a plasmid, pFST-porB-HD (Peng et al., 2017), carrying active gRNA targeting *porB* and two homologous arms of −1 kb each, was electroporated into *C. glutamicum* ATCC13032 harboring the plasmid pXMJ19-SpRY-HF1. Although *Sp*RY-HF1 exhibited high expression (Supplementary Figure S1A), it indicated a exceeding low counter-selection efficiency (Supplementary Figure S1B), which may be attributed to the excessive number of mutation sites that tend to reduce nuclease activity (Okafor et al., 2019) or increase off-target titration in the genome (Moreb and Lynch, 2021). This results in the extension of the search times for targeted loci, and in turn, leads to a reduction in counter-selection efficiency. Hence, this indicates that the gene editing application of the *Sp*Cas9 variant *Sp*RY-HF1 in *C. glutamicum* still requires further optimization.

### Development and optimization of a hybrid CRISPR-Cas9-Cas12a gene editing platform

*Sp*Cas9 and *Fn*Cas12a recognize different PAM sequences of 5′-NGG-3′ and 5′-TTN-3′ respectively (Jinek et al., 2012; Zetsche et al., 2015). Therefore, incorporating both *Sp*Cas9 and *Fn*Cas12a into a unified system facilitates the broadening of the targeting range. Considering this, 2 *C. glutamicum-E. coli* shuttle plasmids were constructed based on the plasmids pFSC (Peng et al., 2017) and pJYS3 (Jiang et al., 2017): pFSC-Cas12a and pJYS3-Cas9, which carried *the P*_*lacM*_ *-Fncas12a-rrnBT1T2-Spcas9-P*_*tac*_ cassette and were transformed into *C. glutamicum* ATCC 13032 (i.e., 13032). However, this yielded no transformants (Supplementary Figure S1B). It has been reported that constitutive expression of plasmid-borne dCas9 from *S. pyogenes* in *C. glutamicum* has proven to be unattainable (Cleto et al., 2016). Thus, it was speculated that this may be due to the oversized plasmid vector and the simultaneous expression of the two Cas nucleases, resulting in a superposition of toxicity. To address these challenges, the *P*_*tac*_-*SpCas9-rrnBT1T2* and *P*_*lacM*_-*Fncas12a-rrnBT1T2* cassettes were integrated into the 13032 genome at the *putA* and *ldh* gene loci, resulting in strains Ptac-9 and L12, respectively (Supplementary Figure S2A). The strains with two Cas-coding gene copies were then constructed to obtain the following: 9–12 with one copy each of *P*_*tac*_-*Spcas9-rrnBT1T2* and *P*_*lacM*_-*cas12a-rrnBT1T2*, Cas9-2 with two copies of *P*_*tac*_-*Spccas9-rrnBT1T2*, and Cas12a-2 with two copies of *Fncas12a* (Supplementary Figure S2A; Supplementary Table S3). In this growth experiment, it was observed that by integrating a single copy of the *P*_*tac*_-*Spcas9-rrnBT1T2* and *P*_*lacM*_-*Fncas12a-rrnBT1T2* into the genome, the growth of strain 13032 was hardly affected, whereas, growth was significantly inhibited when two copies of the *P*_*tac*_-*Spcas9-rrnBT1T2* and *P*_*lacM*_-*Fncas12a-rrnBT1T2* were integrated (Supplementary Figure S2B). Hence, nuclease-induced toxicity can be entirely removed in the 13032 when genomic integration uses a single copy of the *P*_*tac*_-*Spcas9-rrnBT1T2* and *P*_*lacM*_-*Fncas12a-rrnBT1T2*.

**FIGURE 1:**
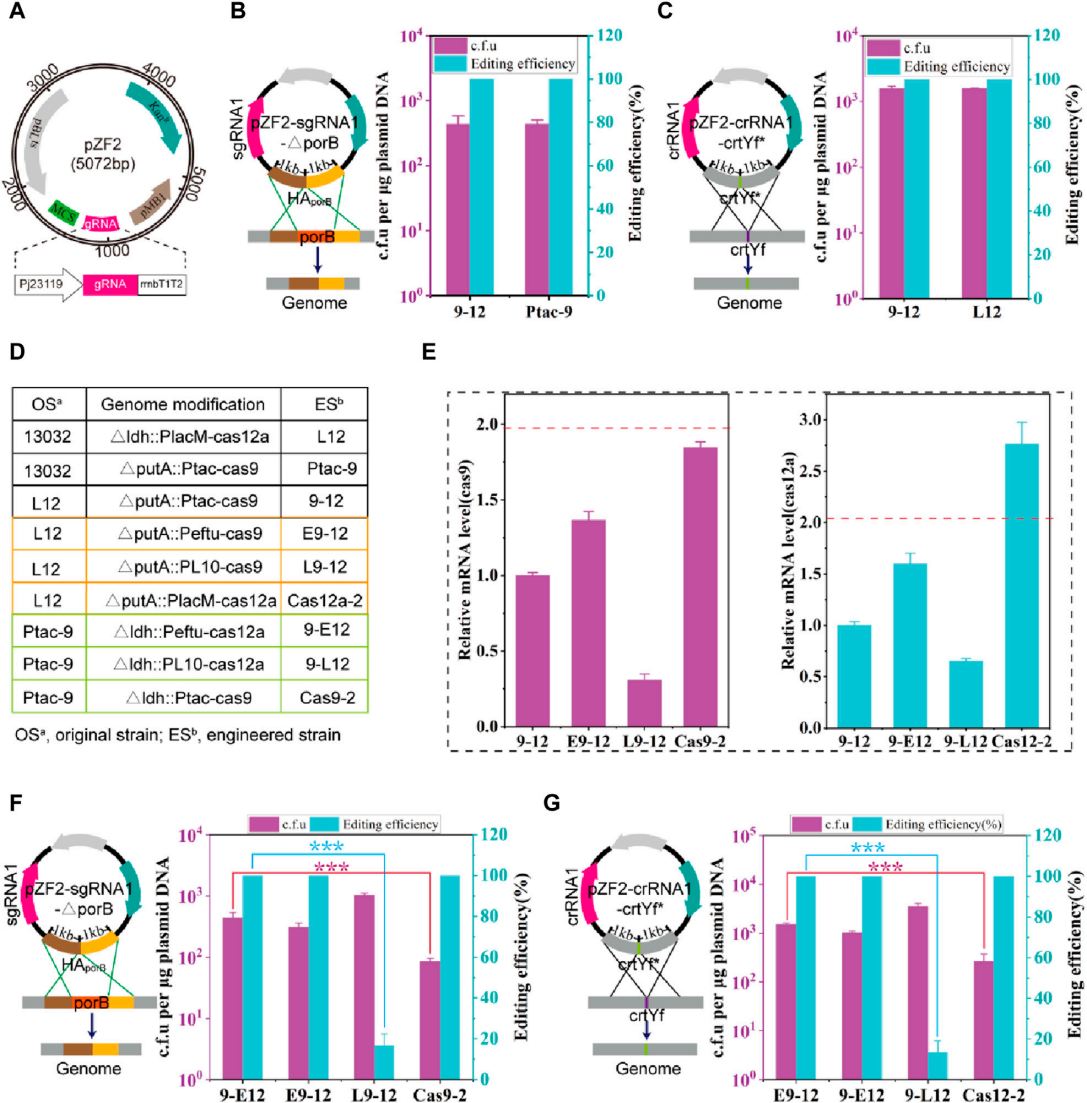
Construction and optimization of HyCas9-12aGEP. **(A)** Schematic diagram of the temperature-sensitive gRNA expression plasmid pZF2. pBL1^ts^: a temperature sensitive replication derived from the pBL1 replicon of *C. glutamicum*; Kn^r^: kanamycin resistance gene encoded by the aminoglycoside phosphotransferase gene; pMB1: a replication origin of *E. coli*; Pj23119: a synthetic constitutive expression promoter. MCS: multiple cloning site, which can be used to assemble homologous repair templates; gRNA: guid RNA; rrnBT1T2: rrnBT1T2 terminator; In this study, sgRNA and crRNA specifically refer to the guide RNAs for *Sp*Cas9 and *Fn*Cas12a, respectively. **(B)** Deletion of a 0.5 kb DNA fragment (*porB*) using the plasmid pZF2-sgRNA1-ΔporB in strains 9-12 and Ptac-9. Plasmid pZF2-sgRNA1-ΔporB expresses sgRNA1 targeting the *porB* locus and carries a 1 kb homologous arm. **(C)** Introduction of 20 nucleotide changes using the plasmid pZF2-crRNA1-crtYf* in strains 9-12 and L12. Plasmid pZF2-crRNA1-crtYf* expresses crRNA1 targeting the *crtYf* locus and carries a 1 kb homologous arm. In the genomes of Ptac-9 and L12 strains, a single copy of the *Spcas9* and *Fncas12a* genes is respectively present. However, the 9–12 strain simultaneously carries a copy of both the *Spcas9* and *Fncas12a* genes. **(D)** Control of the expression of *Spcas9* or *Fncas12a* genes integrated into the genome through different strength promoters and copy numbers. PlacM: synthetic medium-strength promoter; Ptac: synthetic medium-strength promoter; Peftu: strong promoter; PL10: weak promoter (Yim et al., 2013). **(E)** The relative transcription level of *Spcas9* and *Fncas12a* gene. The transcription level of *Sp*cas9 in E9-12, L9-12 and Cas9-2 was compared against that of *Sp*cas9 in 9–12; The transcription level of *Fn*cas12a in 9-E12, 9-L12 and Cas12-2 was compared against that of cas12a in 9–12. **(F)** Impact of *Spcas9* gene expression at different strengths on the efficiency of *porB* deletion. **(G)** Impact of *Fncas12a* gene expression at different strengths on the efficiency of introducing 20 nucleotide substitutions in *crtYf*. Data are presented as mean values+/−SD (*n* = 3 independent experiments). ***p < 0.01, Student’s two-tailed *t*-test. Source data underlying Supplementary Figure S3 are provided as a Source Data file.

Next, the genome-editing performance of the hybrid CRISPR-Cas9-Cas12a system was tested. A temperature-sensitive plasmid, pZF2, was initially designed for expressing the gRNA and harboring homologous arms (HA) (Figure 1A). It has been reported that crRNA-crtYf (Jiang et al., 2017) (crRNA1) and sgRNA-porB (Peng et al., 2017) (sgRNA1) facilitate highly effective gene editing. Thus, crRNA1 and sgRNA1 are assembled into plasmids pZF2 harboring the corresponding homologous arms, obtaining plasmids pZF2-sgRNA1-**△**porB and pZF2-crRNA1-crtYf*, which are used to knock out the gene *porB* (0.5 kb) and introduce point mutations at the *crtYf* site, respectively. The two plasmids were then transformed to 9–12 strain, and the colony PCR results showed that the knockout of *porB* and the point mutation efficiency of *crtYf* reached 100% (Figures 1B,C; Supplementary Figure S3), which was comparable to the optimal editing efficiency previously reported (Jiang et al., 2017; Peng et al., 2017). The strains that were successfully edited were subsequently cultured overnight at 37°C, resulting in plasmid curing efficiencies of approximately 100% (Supplementary Figure S4).

The expression level of Cas proteins is a critical factor influencing editing efficiency (Hsu et al., 2013; Pattanayak et al., 2013). Thus, various strategies, including the replacement of Cas gene promoters and increasing copy numbers (Figure 1D), were employed to optimize the expression levels of Cas genes. A series of *C. glutamicum* carrying the HyCas9-12aGEP were developed, utilizing either a strong P_*eftu*_ promoter or a weak P_*L10*_ (Yim et al., 2013) promoter to regulate the expression of *Spcas9* and *Fncas12a* (Figure 1D). As expected, the transcription levels of *Spcas9* and *Fn*cas12a were consistent with the strength of their respective promoters (Figure 1E). Notably, compared to 9-12, the transcription levels of *Fn*cas12a and *Sp*cas9 in Cas12a-2 and Cas9-2 were increased by 176% and 84%, respectively (Figure 1E), suggesting that the location of genes in the genome affects the expression of genes (Akhtar et al., 2013; Bryant et al., 2014; Goormans et al., 2020). Transformation with 1 μg of pZF2-sgRNA1-△porB into L9-12, produced more than 10^3^ c.f.u, among which −16.6% were correctly edited (Figure 1F). However, compared to 9-E12, Cas9-2 exhibited similar editing efficiency but a significantly reduced number of transformants (Figure 1F). Similar results were observed when the plasmid pZF2-crRNA1-crtYf* was transformed into strains containing cas12a genes controlled by promoters of varying strengths (Figure 1G). These results indicate that insufficient expression levels of *Sp*Cas9 and *Fn*Cas12a result in a high escape rate that is inadequate for eliminating wild-type cells, while excessively high expression levels of *Sp*Cas9 and *Fn*Cas12a lead to reduced transformation efficiency. Therefore, the 9–12 strain, which balances transformation efficiency and editing efficiency, was selected for subsequent experiments.

### HyCas9-12aGEP improves genome editing resolution

Due to the heterogeneity of gRNA activity, there are instances where no active gRNA is available for specific gene loci, leading to a decrease in the resolution of gene editing. For example, CRISPR-*Fn*Cas12a (Jiang et al., 2017) and *Sp*Cas9 (Zhang et al., 2020) have been reported to lack suitable gRNAs for specific gene loci in *proB* and *zwf*, respectively, resulting in failures to precisely introduce mutations at the target gene sites in *C. glutamicum*. Thus, this generally lowers the resolution of CRISPR-Cas-based gene editing compared to the theoretical value. However, by leveraging both *Sp*Cas9 and *Fn*Cas12a nucleases, each recognizing different PAM sequences, HyCas9-12aGEP increases gRNA availability, thereby enhancing gene editing resolution.

**FIGURE 2:**
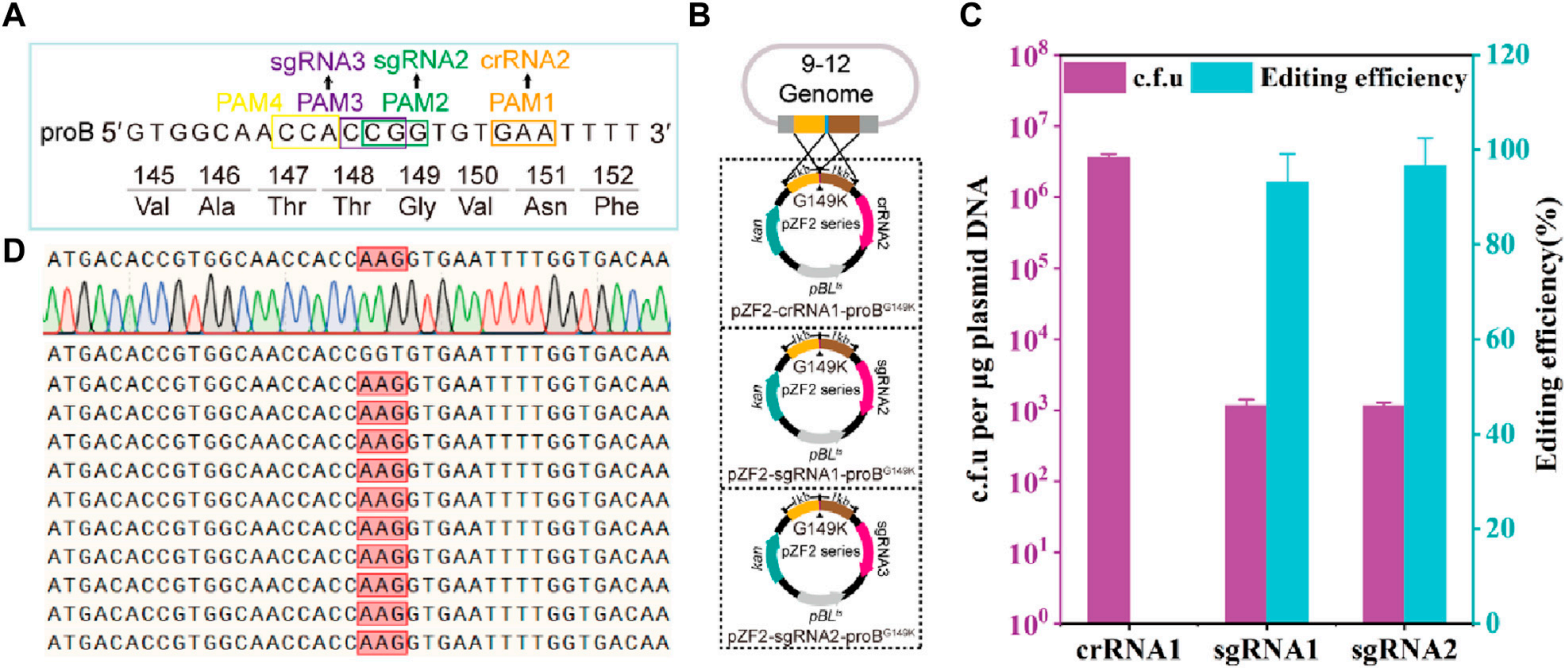
HyCas9-12aGEP improves genome editing resolution. **(A)** DNA sequence near the 149th amino acid residue of the *proB* gene, encoding γ-glutamyl kinase. PAM1 represents the recognition sites for Cas12a/crRNA2; PAM2 and PAM3 are the recognition sites for Cas9/sgRNA2 and Cas9/sgRNA3, respectively. **(B)** Schematic representation of precise mutagenesis at the 149th amino acid residue of the *proB* gene, mediated by crRNA2, sgRNA2, and sgRNA3. A single plasmid carrying a 1 kb homologous arm and the gRNA was transformed into the 9–12 strain. Cas9 and Cas12a/gRNA induce target site cleavage, and the homologous arm with inactivated PAM or seed sequence mutations serves as a template for homologous recombination repair, leading to the precise introduction of an amino acid replacement at the 149th position in ProB. **(C)** Gene editing assessment of the proB (G149K) mutation mediated by crRNA2, sgRNA2, and sgRNA3 in the 9–12 strain. **(D)** Ten representative *proB* recombinants identified by PCR were further sequenced, which revealed the substitution of GGT by AAG for all samples, as expected. Data are presented as mean values+/−SD (*n* = 3 independent experiments). Source data for Supplementary Figure S5 are provided as a Source Data file.

The γ-glutamyl kinase encoded by *proB* is the rate-limiting enzyme in proline synthesis and is subject to feedback inhibition by proline (Perez-Arellano et al., 2006; Wendisch, 2014). Using CRISPR-Cas12a-assisted genome editing, the mutant strain 13032ProB^G149K^ was generated to alleviate the feedback inhibition of proline, involving the introduction of adjacent synonymous mutations (Jiang et al., 2017). However, it has been reported that synonymous mutations do not alter the encoded protein but can influence gene expression (Kudla et al., 2009). Thus, to prevent the introduction of synonymous mutations, theoretically, only 1 PAM is available for FnCas12a to introduce site-directed mutations at the 149th amino acid residue of ProB, while SpCas9 has 3 available PAMs (Figure 2A). Here, crRNA2, sgRNA2 and sgRNA3 are inactivated by mutation of the 149th amino acid residue glycine (GGT) of *Cg*ProB in the homologous arm to lysine (AAG). Thus, the plasmids pZF2-crRNA2-ProB^G149K^, pZF2-sgRNA2-ProB^G149K^, and pZF2-sgRNA3-ProB^G149K^ are created by assembling crRNA2, sgRNA2, sgRNA3 and homology arms into the plasmid pZF2 (Figure 2B). The *Fn*Cas12a-based edited plasmid pZF2-crRNA2-ProB^G149K^ was electroporated into the 9–12 strain, and no transformants containing the ProB^G149K^ mutation were detected (Figure 2C), potentially attributed to insufficient crRNA2 activity (Figure 2C). However, *Sp*Cas9-based editing plasmids pZF2-sgRNA2-ProB^G149K^ and pZF2-sgRNA3-ProB^G149K^ were transformed into the 9–12 strain, produced −1.1×10^3^ c.f.u, among which −93.3% and 96.6% were correctly edited, respectively (Figures 2C; Supplementary Figure S5). The mutated transformants were subsequently further verified by sequencing (Figure 2D). Thus, these results suggest that the HyCas9-12aGEP enables more precise mutations than the CRISPR-Cas12a system, thereby improving gene editing resolution. Furthermore, these findings emphasize the importance of active gRNAs in successful gene editing.

### HyCas9-12aGEP improves the mapping efficiency of active gRNAs

The efficiency of CRISPR-based gene editing is highly dependent on gRNA activity (Figure 2C). Therefore, the selection and design of efficient gRNAs is essential to improve gene editing efficiency. Although tools have been developed to predict gRNA activity in certain hosts, these programs still have limitations in accurately predicting gRNA activity in diverse hosts because different cells and host types are also factors affecting gRNA activity (Moreb and Lynch, 2021). Literature mining is an effective strategy, but the targets of gRNA used in the literature are limited, which is difficult to meet the requirements of different experimental content. Therefore, efficiently mapping active gRNA remains a bottleneck in CRISPR-Cas gene editing experiments.

**FIGURE 3:**
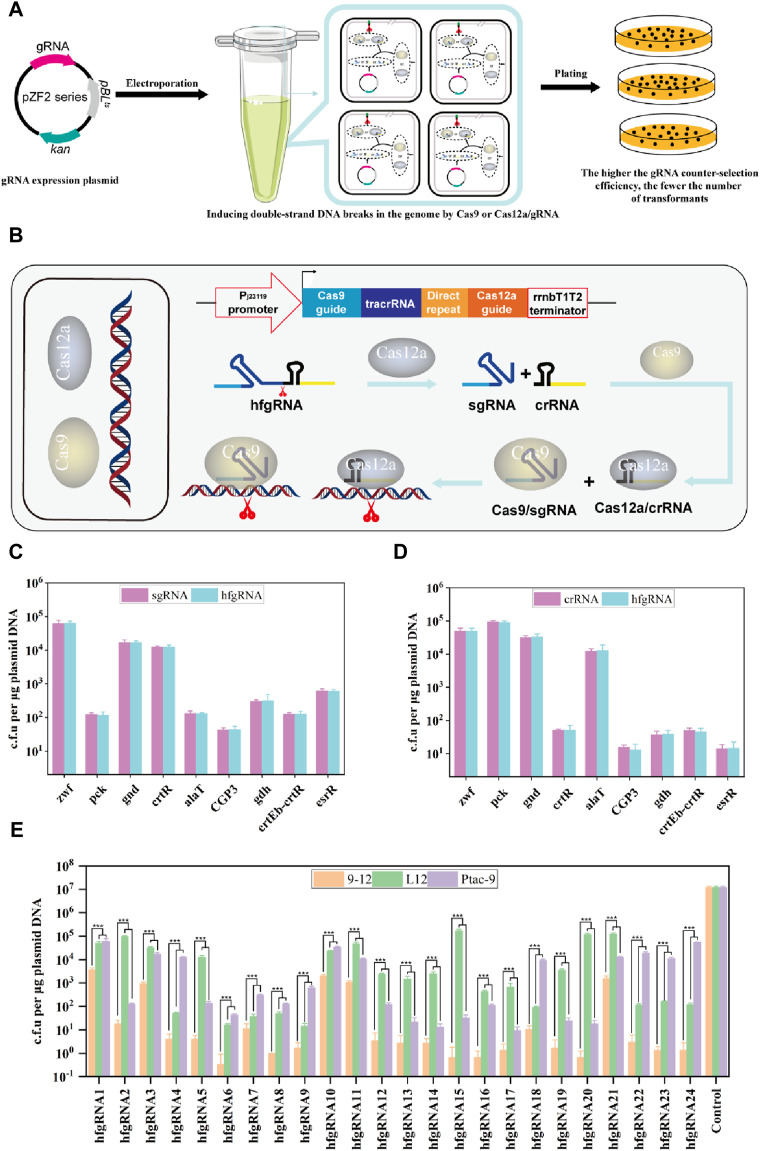
Determination of gRNA activity through experimental methods. **(A)** Schematic representation of gRNA activity assessment based on experiments. I: Plasmids carrying gRNAs were transformed into Ptac-9 (CRISPR-Cas9), L12 (CRISPR-Cas12a), and 9-12 (HyCas9-12aGEP); II: Expressed gRNAs guide Cas9 or Cas12a for genome cleavage; III: The more thorough the cleavage of genomic DNA by Cas9/gRNA or Cas12a/gRNA, the fewer the number of transformants. **(B)** Schematic representation of dual-target DNA cleavage mediated by hfgRNA in 9–12. Transformed hfgRNA is processed by Cas12a to yield crRNA and sgRNA, guiding Cas12a and Cas9 to simultaneously target two genomic loci. **(C, D)** Impact of hfgRNA design on the activity of sgRNAs and crRNAs targeting the *zwf*, *pck*, *gnd*, *crtR*, *alaT*, CGP3, *gdh*, *crtEb*-*crtR* and *esrR* loci. Plasmids expressing sgRNA, crRNA, and their respective fusion hfgRNA were separately transformed into Ptac-9 and L12 to determine the number of transformants. **(E)** Determination of the transformation efficiency of 24 hfgRNAs in Ptac-9, L12, and 9-12. The 24 corresponding target sites for hfgRNAs are provided in Supplementary Data S2. hfgRNA1-9 are used in panels **(C)** and **(D)**. Data are presented as mean values+/−SD (*n* = 3 independent experiments). ***p < 0.01, Student’s two-tailed *t*-test.

Next, we attempted to establish a method for evaluating gRNA activity through experimental assays. It is widely recognized that non-homologous end joining (NHEJ) and homologous recombination (HR) are the primary repair pathways for DNA double-strand breaks. However, it should be noted that the NHEJ pathway is impaired in *C. glutamicum* (Resende et al., 2011) (https://www.kegg.jp/kegg/pathway.html). When gRNA guides *Sp*Cas9 or *Fn*Cas12a to cleavage DNA, in the absence of a homologous repair template, the organism undergoes cell death since the double-stranded DNA break cannot be repaired. Therefore, gRNA activity can be assessed through the transformation efficiency of gRNA expression plasmids lacking homologous repair templates. In other words, higher gRNA activity leads to more efficient SpCas9/gRNA DNA cleavage, resulting in fewer corresponding transformants, and *vice versa* (Figure 3A). Since *Fn*Cas12a can process its own crRNA, transcribed hfgRNA can be processed into independent crRNA and sgRNA, which can orthogonally guide *Sp*Cas9 and *Fn*Cas12a to target two genomic loci (Figure 3B). This allows a single hfgRNA to assess the targeting activity of two designed gRNAs, doubling the efficiency of identifying active gRNAs. As a proof of concept, nine crRNAs and sgRNAs targeting the *zwf*, *pck*, *gnd*, *crtR*, *alaT*, phage CGP3, *gdh*, *crtEb-crtR* and *esrR* loci, were transcribed individually or co-transcribed with their respective nine hfgRNAs. Unexpectedly, we did not observe a significant difference in the counter-selection efficiency of hfgRNA in Ptac-9 and L12 when compared to independently transcribed sgRNA and crRNA (Figures 3C,D). These results suggested that hfgRNA design does not affect the activity of sgRNA or crRNA, thereby confirming that HyCas9-based approaches can indeed improve the efficiency of identifying active gRNAs. Notably, the counter-selection efficiency achieved with hfgRNA in strain 9–12 exhibited a significant enhancement compared to Ptac-9 and L12 (Figure 3E). Similarly, based on the gRNA online design tool (http://www.rgenome.net/cas-designer/), we designed 15 crRNAs and 15 sgRNAs and assembled them into corresponding hfgRNAs (Supplementary Data S1, S2), which can also significantly improve the counter-selection efficiency in 9–12 (Figure 3E). These results suggested that hfgRNA based on HyCas9-12aGEP can also improve the counter-selection efficiency.

### HyCas9-12aGEP combined with hfgRNA improves the genome editing efficiency

**FIGURE 4:**
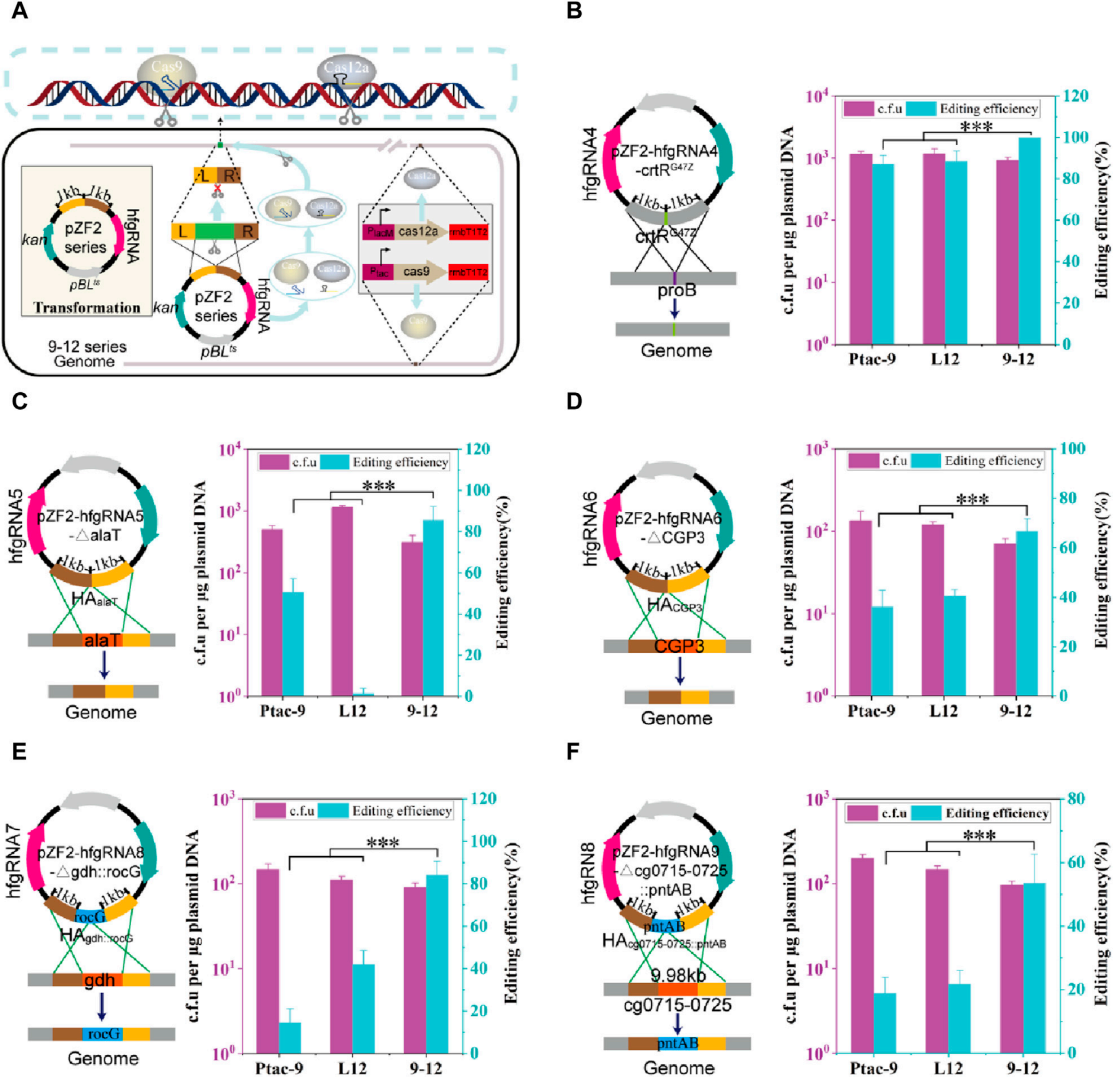
**G**enome editing mediated by hfgRNA in CRISPR-Cas9, CRISPR-Cas12a, and HyCas9-12aGEP. **(A)** Schematic representation of gene editing mediated by hfgRNA in the HyCas9-12aGEP system. The transcribed hfgRNA is processed by *Fn*Cas12a to generate crRNA and sgRNA, which respectively guide *Fn*Cas12a and *Sp*Cas9 to simultaneously target and cleave two distinct loci in the genome. Homologous recombination is employed for the introduction of the desired mutations. **(B–F)** Genome editing using hfgRNA in the 9–12 strain harboring HyCas9-12aGEP. hfgRNA4-8 are designed to target *crtR*, *alaT*, CGP3, *gdh*, and *crtR/crtEb*, for the introduction of mutations including crtRG47Z, ∆alaT (0.5 kb), ∆CGP3 (219 kb), ∆gdhrocG, and ∆cg0715-0725pntAB. CrtR^G47Z^ involves the substitution of the 47th glycine residue in CrtR with a stop codon to deactivate the *crtR* gene. ∆alaT (0.5 kb): deletion of a 0.5 kb segment of the *alaT* gene. ∆CGP3 (219 kb): the complete removal of the CGP3 bacteriophage, spanning 219 kb ∆gdhrocG: deletion of the endogenous glutamate dehydrogenase gene *gdh* (1.34 kb) and concomitant insertion of the expression cassette Ptac-rocG-rrnBT1T2 (1.58 kb), encoding the glutamate dehydrogenase rocG derived from *Bacillus subtilis*. ∆cg0715-0725pntAB: deletion of the gene cluster cg0715-0725 while inserting the expression cassette PH36-pntAB-rrnBT1T2 (3.5 kb) from *E. coli*. Data are presented as mean values+/−SD (*n* = 3 independent experiments). ***p < 0.01, Student’s two-tailed *t*-test. Source data underlying Supplementary Figure S6 are provided as a Source Data file.

Based on HyCas9-12aGEP, hfgRNA effectively improves the counter-selection efficiency (Figure 3E). To test hfgRNA gene editing performance, plasmids expressing hfgRNA and harboring two −1 kb HAs were transformed into 9–12, Ptac-9, and L12 strains (Figure 4A). First, hfgRNA4 was used to introduce a stop codon into the *crtR* gene to inactivate CrtR (CrtR^G47Z^). Although the site-directed mutation efficiency of hfgRNA4 in Ptac-9 and L12 is as high as 86.9% and 88.4%, respectively, the editing efficiency reached 100% in 9–12 (Figure 4B). Similarly, when hfgRNA5 was used to delete the 0.5 kb *alaT* gene in 9–12, an editing efficiency of 85.5% was obtained, which was significantly higher than the 50.7% and 1.44% obtained in Ptac-9 and L12 (Figure 4C). Notably, when hfgRNA6 was employed in Ptac-9 and L12 to delete a 219 kb DNA fragment (intact phage CGP3), editing efficiencies of 36.2% and 40.6% were achieved, respectively, slightly exceeding the 34.8% efficiency previously reported (Liu et al., 2022) (Figure 4D). However, when the CGP3 (219 kb) was deleted in strains 9–12, an editing efficiency of 66.6% was achieved, surpassing the levels attainable in Ptac-9 and L12 (Figure 4D).

Next, we tested the editing efficiency of hfgRNA in insertion. To optimize chemical and biofuel production, strategies in cofactor engineering have been devised, including adjustments in cofactor supply and modifications to reactants’ cofactor preference, ensuring redox balance (Wang et al., 2017). Thus, hfgRNA7 was used to delete the NADPH-dependent glutamate dehydrogenase gene *gdh* (1.34 kb) deletion in *C. glutamicum*, while insert the NADH-dependent glutamate dehydrogenase gene *rocG* (1.58 kb) from *Bacillus subtilis* 168. The editing efficiency of hfgRNA5 in Ptac-9 and L12 was 14.5% and 42.0%, respectively, but it was as high as 84.0% in 9–12 (Figure 4E). Finally, we also test whether hfgRNA8 can achieve deletion of large fragments (9.98 kb) while inserting the *E. coli*-derived *pntAB* (3.5 kb) operon. The membrane-bound transhydrogenase encoded by *pntAB* is advantageous for enhancing the supply of NADPH, thereby promoting the synthesis of high-value metabolic products (Kleine et al., 2017). Notably, hfgRNA6 achieved a deletion-insertion efficiency of 53.6% in 9–12 strain, significantly higher than Ptac-9 and L12, which yielded efficiencies of 18.8% and 21.7%, respectively (Figure 4F). In conclusion, the above results suggested that hfgRNA significantly improves the efficiency of genome editing based on HyCas9-12aGEP.

### Application of the HyCas9-12aGEP for efficient phenotype-genotype mapping

**FIGURE 5:**
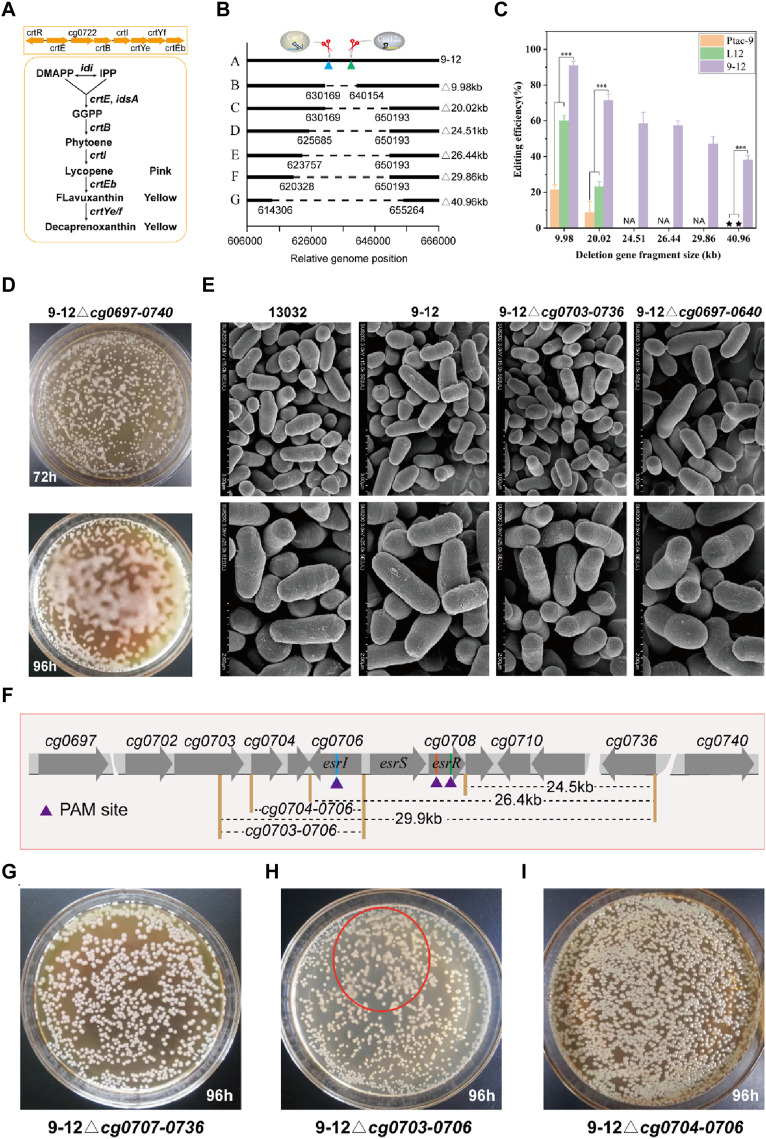
Application of HyCas9-12aGEP for phenotype-genotype mapping via large DNA segment deletion. **(A)** The reaction catalyzed by the *crtEBIYe/fEb* gene cluster for terpenoid biosynthesis. DMAPP: dimethylallyl pyrophosphate; IPP: isopentenyl pyrophosphate; GGPP: geranylgeranyl diphosphate; **(B)** Relative genomic positions of *cg0725-0725* (9.98 kb), *cg0715-0736* (20.02 kb), *cg0710-0736* (24.51 kb), *cg0707-0736* (26.44 kb), *cg0703-0736* (29.86 kb), and *cg0697-0740* (40.96 kb) in *C. glutamicum* ATCC13032. **(C)** Deletion efficiency of cg0725-0725 (9.98 kb), cg0715-36 (20.02 kb), cg0710-0736 (24.51 kb), cg0707-0736 (26.44 kb), cg0703-0736 (29.86 kb), and cg0697-0740 (40.96 kb) guided by hfgRNA8. **(D)** Phenotypic changes induced by the deletion of cg0697-0740 (40.96 kb) in *C. glutamicum*. Deletion of cg0697-0740 (40.96 kb) leads to increased ‘moisture’ of *C. glutamicum* on CM agar plates. **(E)** Scanning electron microscopy results of five different genotypes of **(C)** *glutamicum*, including 13032, 9-12, 9-12△cg0703-0736, and 9-12△cg0697-0740. **(F)** Phenotype-genotype mapping strategy. Rapid phenotype-genotype mapping is achieved through a strategy of halving the number of genes each time to pinpoint the genotypes responsible for the observed phenotypic changes. **(G–I)** Colony phenotypes of 9–12△cg0707-0736, 9-12△cg0704-0706, and 9-12△cg0703-0706 on CM agar plates after 96 h, respectively. The red ellipse indicates the corresponding ‘moisture’ colony phenotype observed in 9–12△cg0703-0706. Data are presented as mean values+/−SD (*n* = 3 independent experiments). ***p < 0.01, Student’s two-tailed *t*-test. Source data underlying Supplementary Figure S7 and Supplementary Table S3 are provided as a Source Data file.

Large fragment genome editing can be used to study the function of genes and noncoding regions. By deleting large DNA fragments, researchers can gain profound insights into how these regions impact an organism’s growth, development, and physiological processes. The complete *crtREBIYe/fEb* gene cluster (*cg0717-0725*) is an important factor in the yellow color of *C. glutamicum*, and deletion of the *cg0717-0725* may cause the color of the organism to change (Figure 5A). Here, hfgRNA8 was used to performed large fragment gene deletion testing of *cg0715-0725* (9.98 kb), *cg0715-0736* (20.02 kb) and *cg0697-cg0740* (40.96 kb) (Figure 5B). For deletion of *cg0715-0725*, transformants of two colony morphologies, white and yellow, were obtained (Supplementary Figure S7A). The white single clones were picked for PCR verification, and the results showed that they were all successfully edited strains (Supplementary Figure S7B). Hence, the success of editing can be determined by the color of individual colonies (Supplementary Figures S7B, S8A). As expected, hfgRNA8 significantly improved editing efficiency in 9–12 compared to Ptac-9 and L12 (Figure 5C; Supplementary Table S3). Remarkably, the deletion of 40.9 kb failed to yield detectable successfully edited strains in L12 and Ptac-9, whereas 9–12 maintained a remarkable editing efficiency of 38.1%. Unexpectedly, the deletion of the 40.9 kb fragment altered the colony morphology, rendering it more “moisture” (Figure 5D). However, no significant changes in the morphology of the bacteria were found by electron microscopy (Figure 5E). Therefore, we inferred that this phenotype may be caused by significant metabolic changes.

Next, we performed gradual gene fragment deletions to identify phenotype-influencing genes (Figure 5F). Since the expected phenotype didn't emerge with the 20 kb gene fragment knockout, the gene affecting the phenotype likely resides in the 20–40 kb region. Deleting the 29.86 kb DNA fragment (47.2% editing efficiency) revealed the phenotype (Supplementary Figure S8B), pinpointing the gene affecting it in the 20–30 kb range. Notably, the three-component system EsrISR (encoded by *cg0706-0708*) regulates a cell envelope stress response in *C. glutamicum* (Kleine et al., 2017). However, sequential deletions of *cg0710-0736* (24.51 kb) and *cg0707-0736* (26.44 kb) fragments did not induce the same phenotype (Figure 5G), which suggested that the EsrISR three-component system has an extremely limited contribution to this phenotype, and deletion experiments of *cg0706-0708* using hfgRNA9 also support this finding (Supplementary Figures S8C, D). Thus, only *cg0703-0706* remained unevaluated. Finally, crRNA11 was employed to target *esrI* (*cg0706*) and facilitate the deletion of *cg0703-0706*, resulting in the strain 9–12△cg0703-0706 (Supplementary Figure S8D), which exhibited a milder version of the phenotype compared to the 29.86 kb and 40.96 kb deletions (Figure 5H). Moreover, 9-12△cg0704-0706 strain did not yield the expected phenotype (Figure 5I; Supplementary Figure S8D). Thus, it can be inferred that the deletion of *cg0703* is responsible for this phenotype, but the deletion of *cg0704-0736* also greatly contributed to it. The precise regulatory mechanism can be further elucidated through subsequent transcriptomic and metabolomic analyses. In conclusion, based on HyCas9-12aGEP, hfgRNA enables efficient phenotype-genotype mapping.

### Applicability of HyCas9-12aGEP in other *Corynebacterium* species

**FIGURE 6:**
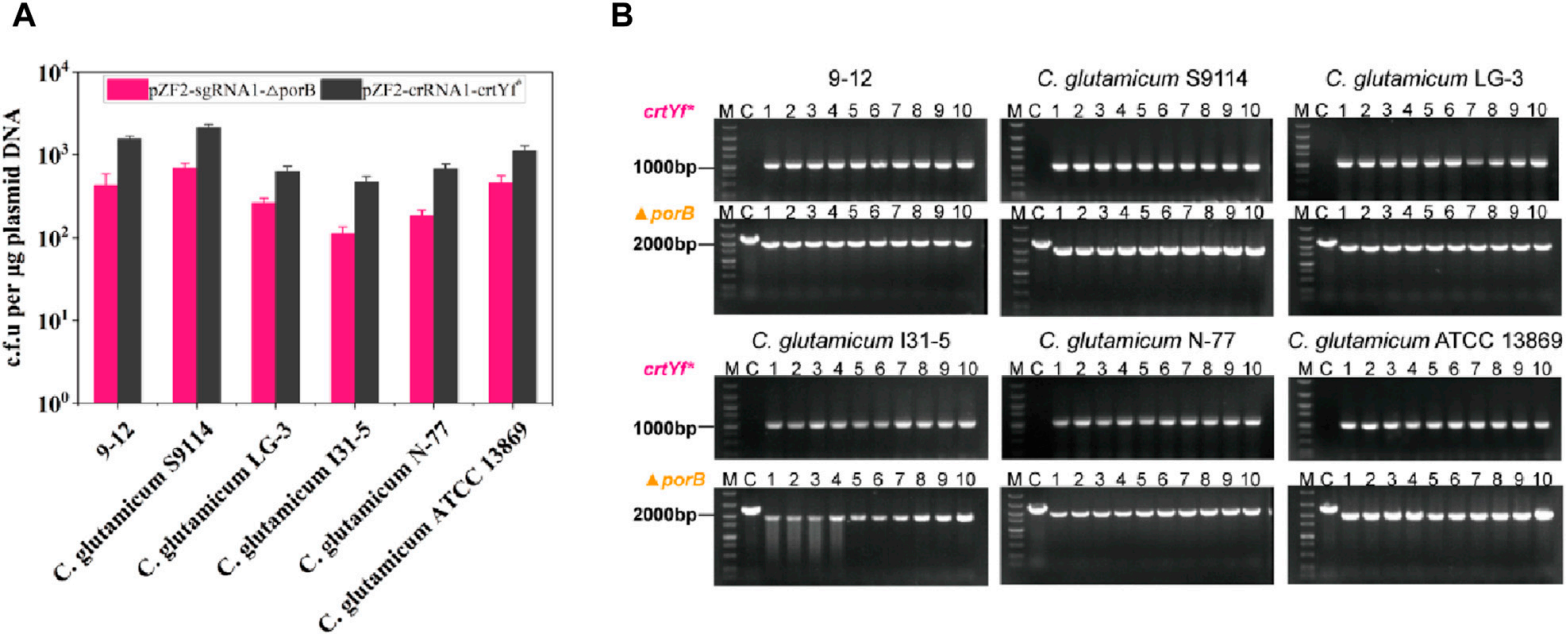
Applications of HyCas9-12aGEP in other *Corynebacterium* species. **(A)** Growth of *Corynebacterium* strains carrying the integrated CRISPR-Cas9-Cas12a system transformed with pZF2-sgRNA1-△porB and pZF2-sgRNA1-crtYf*. Experiments were performed in duplicates. **(B)** Ten transformant colonies of the indicated *Corynebacterium* strains derived from pZF2-sgRNA1-△porB and pZF2-sgRNA1-crtYf*-based HyCas9-12aGEP recombining were analyzed by colony PCR. A DNA ladder (DL 5000 DNA Marker, Vazyme Biotech Co.,Ltd) was used as a marker.

To determine whether HyCas9-12aGEP can be constructed in other species of *Corynebacterium* except for strain 13032, the HyCas9-12aGEP was introduced into strains *C. glutamicum* S9114, *C. glutamicum* ATCC 13869, *C. glutamicum* LG-3, *C. glutamicum* N-77, *and C. glutamicum* I31-5 by sequentially integrating the *Cas12a* and *SpCas9* genes at *the ldh* and *putA* locus of these strains genome. Plasmids pZF2-sgRNA1-△porB and pZF2-crRNA1-crtYf* were transformed to a series of strains harboring HyCas9-12aGEP to evaluate the editing efficiency. Although differences in transformation efficiency were observed among different strains harboring HyCas9-12aGEP (Figure 6A), all six tested strains exhibited comparable editing efficiency for the target loci (Figure 6B). Hence, these results suggested that the HyCas9-12aGEP system can be applied to other species of *Corynebacterium*.

### Off target analysis of HyCas9-12aGEP

To analysis the off-target effect in *C. glutamicum* after gene editing by HyCas9-12aGEP, genome resequencing was performed to identify all the single nucleotide polymorphism (SNP) and insertions and deletions (Indel). The strains analyzed included 9-12**△** *porB* (*porB*-deleted strain), 9-12**△** *cg0697-0740* (*cg0697-0740*-deleted strain) and the 9-12**△** *gdh::rocG* (*gdh*-deleted and rocG-inserted strain), with wild-type 13032 serving as the negative control. Furthermore, to investigate whether the *Sp*Cas9 and *Fn*Cas12a protein induce off-target effects in the absence of gRNA, the SNP and Indel profiles of the 9–12 strain harboring *Sp*Cas9 and *Fn*Cas12a proteins were also examined. The results indicated that no off-target mutations were identified in the 9–12 strain containing *Sp*Cas9 and *Fn*Cas12a proteins. That is, in comparison to the wild-type strain, only two Indels (corresponding to *ldh* and *putA* deletions) and two intergenic Indels (corresponding to *Spcas9* and *Fncas12a* integrations) were detected, with no SNPs observed in this strain. Meanwhile, No SNP and Indel were identified in the 9-12△*cg0697-0740* and 9-12△*gdh::rocG* strains (Supplementary Table S4). Notely, compared to previous reports where 1 Indel with 1 base deleted was identified during the resequencing of *porB*-deleted strains (Peng et al., 2017), we did not observe any SNP and Indel in the 9-12△*porB* strains (Supplementary Table S4). The results suggested that the genomic integration of the *SpCas9* gene effectively reduces the off-target effects of *Sp*Cas9.

## Discussion

HyCas9-12aGEP in *C. glutamicum* was developed and optimized to improve the resolution of genome editing. Leveraging hfgRNA design, HyCas9-12aGEP outperforms both CRISPR-Cas9 and CRISPR-Cas12a in terms of gene editing efficiency and active gRNA mapping. HyCas9-12aGEP’s robust capability for large-fragment editing enables rapid identification of genotype-phenotype relationships among over 40 genes. Integrating *Sp*Cas9 expression into the genome effectively minimizes off-target editing. In summary, HyCas9-12aGEP streamlines gene editing in *C*. *glutamicum*, enhancing accessibility and promoting a more sustainable, efficient, and precise biological production process.

SpRY-HF1, an unconstrained near-PAMless and high-fidelity *Sp*Cas9 variant, was inferred to have low nuclease activity in *C. glutamicum* as it is the active gRNA for *Sp*Cas9 but not for SpRY-HF1 and its expression level in *C. glutamicum* is also comparable to *Sp*Cas9. *Sp*Cas9/gRNA exhibits stable DNA binding with just an 8-9 bp match to the PAM-proximal region (Singh et al., 2018). However, the SpRY-Cas9 variant, which is generally unrestricted by PAM sites, indicates that a large number of SpRY-Cas9/sgRNA complexes will be titrated on the numerous homologous sequences in the genome. In effect, there were significant reductions in effective SpRY-Cas9/gRNA complex concentrations, which prolong the time for it to search for the target site. Consequently, this decreases the editing activity of the gRNA (Moreb and Lynch, 2021). Meanwhile, the conformational state of the HNH nuclease domain directly controls the DNA cleavage activity, in which the DNA cleavage efficiency is proportional to the extent of the activated conformation of the HNH domain (Sternberg et al., 2015). For *Sp*Cas9, the HNH-activated conformation is closely linked to the unwinding state of the DNA target, accounting for 78%–100% of the unwinding portion of all DNA targets (Dagdas YS et al., 2017). *Sp*Cas9-HF1 also has a reduced ability to unwind target DNA (Okafor et al., 2019), which inevitably inhibits DNA cleavage activity. The titration effect of SpRY-Cas9 and the reduced cleavage efficiency of high-fidelity *Sp*Cas9-HF1 may have potentially resulted in significant reductions of SpRY-HF1 nuclease activity in *C. glutamicum*.

We attempted unsuccessfully to transform plasmids pFSC-Cas12a and PYJS3-Cas9, which co-express *Sp*Cas9 and *Fn*Cas12a, into *C. glutamicum*. Plasmids expressing dCas9 in constitutive forms have been challenging to transform in *C*. *glutamicum* (Cleto et al., 2016). However, optimizing promoters and ribosomal binding sites (RBS) has significantly improved transformation efficiency by reducing *Sp*Cas9 and *Fn*Cas12a expression (Cleto et al., 2016; Liu et al., 2017; Li et al., 2020). Furthermore, integrating one copy of the *Sp*Cas9 gene into the genome has effectively mitigated the toxicity of *Sp*Cas9 proteins with minimal impact on bacterial growth (Wang et al., 2018). The plasmid copy number is governed by the replicon, with pJYS3 and pFSC plasmids’ replicon, *pBL1*, having a range of 10–30 copies (Pátek and Nešvera, 2013). This elevated *Sp*Cas9 or *Fn*Cas12a expression resulting from the multi-copy *cas* gene could potentially lead to cytotoxicity. Therefore, this explains why a single copy integration of the *Spcas9* or *Fncas12a* gene does not result in cytotoxicity, while having two copies of either *Sp*cas9 or *Fn*cas12a genes does. Although high concentrations of *Sp*Cas9 proteins can induce cytotoxicity (Cleto et al., 2016) and off-target effects (Hsu et al., 2013; Pattanayak et al., 2013), low-level *Sp*Cas9 or *Fn*Cas12a expression reduces editing efficiency (Wang et al., 2018) (Figures 1E,F). Therefore, optimizing the expression levels of *Sp*Cas9 or *Fn*Cas12a proteins is a crucial step in enhancing gene editing efficiency.

The target sites (PAM sites of 5′-NGG-3′ and 5′-TTN-3′) that HyCas9-12aGEP can use are the sum of *Sp*Cas9 and *Fn*Cas12a, thereby improving gene editing resolution compared to CRISPR-Cas9 or Cas12a. Although this study demonstrates that the *Sp*Cas9 system can accurately introduce mutations at amino acid 149 of ProB, *Fn*Cas12a cannot. However, we also found that the gRNA activity of *Sp*Cas9 is diverse in our experiments (Figure 2C, Figures 3C–E), consistent with previous reports (Doench et al., 2014; Moreb and Lynch, 2021; Corsi et al., 2022). In addition, Cas12a has been reported to be more efficient at cutting covalently modified DNA duplexes than Cas9 (Vlot et al., 2018; Dong et al., 2021). Covalent DNA modifications are prevalent across organisms, giving Cas12a an edge over Cas9 when targeting sites within covalently modified genomes. Based on these analyses, the HyCas9-12aGEP improved the resolution of gene editing compared to the CRISPR-Cas9 and Cas12a gene-editing systems. With the discovery of novel Cas proteins (Pausch et al., 2020; Karvelis et al., 2021; Ozcan et al., 2021; Wu et al., 2021; Tsuchida et al., 2022) and application of modified high-performance variants (Kleinstiver et al., 2019; DeWeirdt et al., 2021) in *C. glutamicum*, integration into the genome can further improve gene editing resolution and editing efficiency to build higher-dimensional CRISPR systems.

Based on hfgRNA, HyCas9-12aGEP significantly improves gene editing efficiency compared to classical CRISPR-Cas9 and CRISPR-Cas12a gene editing tools. Previously described CRISPR-Cas9 or Cas12a-based multi-targeting strategies co-expressed pairs of Cas9 (Liu et al., 2017; Horlbeck et al., 2018; Zhao et al., 2018; Zhao et al., 2020) or Cas12a gRNA (Campa et al., 2019; Liu et al., 2019), however, these approaches have limited efficiency, especially in synchronous targeting. Meanwhile, some systems employing orthologous *Staphylococcus aureus* Cas9 and *Sp*Cas9 enzymes (Najm et al., 2017; Boettcher et al., 2018) or *Sp*Cas9 and *Lachnospiraceae bacterium* Cas12a (Gonatopoulos-Pournatzis et al., 2020), like HyCas9-12aGEP, have increased editing efficiency, possibly due to reduced recombination through the use of different transactivating CRISPR RNAs. Taken together, HyCas9-12aGEP greatly improve our capability in terms of genome reprogramming in *C. glutamicum.* Furthermore, we envision that HyCas9-12aGEP is also applicable to other microorganisms.

An efficient gene editing system should not only have reliable and efficient on-target gene editing efficiency, but also should produce minimal off-target effects. It is reported that high levels of Cas9 protein expression would increase the off-target effects (Hsu et al., 2013). In this study, the expression of *Sp*Cas9/*Fn*Cas12a protein was reduced by integrating *Spcas9*/*Fncas12a* gene into genome, which was beneficial to reduce the probability of off-target. Indeed, our resequencing results confirmed that HyCas9-12aGEP did not induce off-target editing at the previously identified *porB* off-target sites (Peng et al., 2017). Although the use of hfgRNA in HyCas9-12aGEP for gene editing theoretically raises the probability of off-target effects, our resequencing results for 9-12△*porB*, 9-12△*cg0697-0740* and 9-12△*gdh::rocG* strains did not reveal any off-target editing. In the further research, it will theoretically contribute to reducing the potential for off-target effects by replacing the high-fidelity Cas9 and Cas12a variants (Chen et al., 2017).

## Data Availability

The datasets presented in this study can be found in online repositories. The names of the repository/repositories and accession number(s) can be found in the article/Supplementary Material.

## References

[B1] Abdel-Mawgoud A. M. Stephanopoulos G. (2020). Improving CRISPR/Cas9-mediated genome editing efficiency in *Yarrowia lipolytica* using direct tRNA-sgRNA fusions. Metab. Eng. 62, 106–115. 10.1016/j.ymben.2020.07.008 32758536

[B2] Akhtar W. de Jong J. Pindyurin A. Pagie L. Meuleman W. de Ridder J. (2013). Chromatin position effects assayed by thousands of reporters integrated in parallel. Cell 154, 914–927. 10.1016/j.cell.2013.07.018 23953119

[B3] Becker J. Rohles C. M. Wittmann C. (2018). Metabolically engineered *Corynebacterium glutamicum* for bio-based production of chemicals, fuels, materials, and healthcare products. Metab. Eng. 50, 122–141. 10.1016/j.ymben.2018.07.008 30031852

[B4] Boettcher M. Tian R. Blau J. A. Markegard E. Wagner R. T. Wu D. (2018). Dual gene activation and knockout screen reveals directional dependencies in genetic networks. Nat. Biotechnol. 36, 170–178. 10.1038/nbt.4062 29334369 PMC6072461

[B5] Bryant J. A. Sellars L. E. Busby S. J. Lee D. J. (2014). Chromosome position effects on gene expression in *Escherichia coli* K-12. Nucleic Acids Res. 42, 11383–11392. 10.1093/nar/gku828 25209233 PMC4191405

[B6] Campa C. C. Weisbach N. R. Santinha A. J. Incarnato D. Platt R. J. (2019). Multiplexed genome engineering by Cas12a and CRISPR arrays encoded on single transcripts. Nat. Methods 16, 887–893. 10.1038/s41592-019-0508-6 31406383

[B7] Chen J. S. Dagdas Y. S. Kleinstiver B. P. Welch M. M. Sousa A. A. Harrington L. B. (2017). Enhanced proofreading governs CRISPR-Cas9 targeting accuracy. Nature 550, 407–410. 10.1038/nature24268 28931002 PMC5918688

[B8] Chen X. Rinsma M. Janssen J. M. Liu J. Maggio I. Gonçalves M. A. (2016). Probing the impact of chromatin conformation on genome editing tools. Nucleic Acids Res. 44, 6482–6492. 10.1093/nar/gkw524 27280977 PMC5291272

[B9] Cleto S. Jensen J. V. K. Wendisch V. F. Lu T. K. (2016). *Corynebacterium glutamicum* metabolic engineering with CRISPR interference (CRISPRi). ACS Synth. Biol. 5, 375–385. 10.1021/acssynbio.5b00216 26829286 PMC4877668

[B10] Cong L. Ran F. A. Cox D. Lin S. Barretto R. Habib N. (2013). Multiplex genome engineering using CRISPR/cas systems. Science 339, 819–823. 10.1126/science.1231143 23287718 PMC3795411

[B11] Corsi G. I. Qu K. Alkan F. Pan X. Luo Y. Gorodkin J. (2022). CRISPR/Cas9 gRNA activity depends on free energy changes and on the target PAM context. Nat. Commun. 13, 3006. 10.1038/s41467-022-30515-0 35637227 PMC9151727

[B12] Creutzburg S. C. A. Wu W. Y. Mohanraju P. Swartjes T. Alkan F. Gorodkin J. (2020). Good guide, bad guide: spacer sequence-dependent cleavage efficiency of Cas12a. Nucleic Acids Res. 48, 3228–3243. 10.1093/nar/gkz1240 31989168 PMC7102956

[B13] Dagdas Ys C. J. Sternberg S. H. Doudna J. A. Yildiz A. (2017). A conformational checkpoint between DNA binding and cleavage by CRISPR-Cas9. Sci. Adv. 3, eaao0027. 10.1126/sciadv.aao0027 28808686 PMC5547770

[B14] DeWeirdt P. C. Sanson K. R. Sangree A. K. Hegde M. Hanna R. E. Feeley M. N. (2021). Optimization of AsCas12a for combinatorial genetic screens in human cells. Nat. Biotechnol. 39, 94–104. 10.1038/s41587-020-0600-6 32661438 PMC7854777

[B15] Doench J. G. Hartenian E. Graham D. B. Tothova Z. Hegde M. Smith I. (2014). Rational design of highly active sgRNAs for CRISPR-Cas9–mediated gene inactivation. Nat. Biotechnol. 32, 1262–1267. 10.1038/nbt.3026 25184501 PMC4262738

[B16] Dong J. Chen C. Liu Y. Zhu J. Li M. Rao V. B. (2021). Engineering T4 bacteriophage for *in vivo* display by type V CRISPR-cas genome editing. ACS Synth. Biol. 10, 2639–2648. 10.1021/acssynbio.1c00251 34546037 PMC12867177

[B17] Gonatopoulos-Pournatzis T. Aregger M. Brown K. R. Farhangmehr S. Braunschweig U. Ward H. N. (2020). Genetic interaction mapping and exon-resolution functional genomics with a hybrid Cas9–Cas12a platform. Nat. Biotechnol. 38, 638–648. 10.1038/s41587-020-0437-z 32249828

[B18] Gong S. Yu H. H. Johnson K. A. Taylor D. W. (2018). DNA unwinding is the primary determinant of CRISPR-cas9 activity. Cell Rep. 22, 359–371. 10.1016/j.celrep.2017.12.041 29320733 PMC11151164

[B19] Goormans A. R. Snoeck N. Decadt H. Vermeulen K. Peters G. Coussement P. (2020). Comprehensive study on *Escherichia coli* genomic expression: does position really matter? Metab. Eng. 62, 10–19. 10.1016/j.ymben.2020.07.007 32795614

[B20] Guo C. Ma X. Gao F. Guo Y. (2023). Off-target effects in CRISPR/Cas9 gene editing. Front. Bioeng. Biotechnol. 11, 1143157. 10.3389/fbioe.2023.1143157 36970624 PMC10034092

[B21] Guo J. Wang T. Guan C. Liu B. Luo C. Xie Z. (2018). Improved sgRNA design in bacteria via genome-wide activity profiling. Nucleic Acids Res. 46, 7052–7069. 10.1093/nar/gky572 29982721 PMC6101607

[B22] Horlbeck M. A. Witkowsky L. B. Guglielmi B. Replogle J. M. Gilbert L. A. Villalta J. E. (2016). Nucleosomes impede Cas9 access to DNA *in vivo* and *in vitro* . Elife 5, e12677. 10.7554/elife.12677 26987018 PMC4861601

[B23] Horlbeck M. A. Xu A. Wang M. Bennett N. K. Park C. Y. Bogdanoff D. (2018). Mapping the genetic landscape of human cells. Cell 174, 953–967. 10.1016/j.cell.2018.06.010 30033366 PMC6426455

[B24] Hsu P. D. Scott D. A. Weinstein J. A. Ran F. A. Konermann S. Agarwala V. (2013). DNA targeting specificity of RNA-guided Cas9 nucleases. Nat. Biotechnol. 31, 827–832. 10.1038/nbt.2647 23873081 PMC3969858

[B25] Ivanov I. E. Wright A. V. Cofsky J. C. Aris K. D. P. Doudna J. A. Bryant Z. (2020). Cas9 interrogates DNA in discrete steps modulated by mismatches and supercoiling. Proc. Natl. Acad. Sci. U. S. A. 117, 5853–5860. 10.1073/pnas.1913445117 32123105 PMC7084090

[B26] Jiang W. Bikard D. Cox D. Zhang F. Marraffini L. A. (2013). RNA-guided editing of bacterial genomes using CRISPR-Cas systems. Nat. Biotechnol. 31, 233–239. 10.1038/nbt.2508 23360965 PMC3748948

[B27] Jiang Y. Qian F. Yang J. Liu Y. Dong F. Xu C. (2017). CRISPR-Cpf1 assisted genome editing of *Corynebacterium glutamicum* . Nat. Commun. 8, 15179–15189. 10.1038/ncomms15179 28469274 PMC5418603

[B28] Jinek M. Chylinski K. Fonfara I. Hauer M. Doudna J. A. Charpentier E. (2012). A programmable dual-RNA–guided DNA endonuclease in adaptive bacterial immunity. Science 337, 816–821. 10.1126/science.1225829 22745249 PMC6286148

[B29] Karvelis T. Druteika G. Bigelyte G. Budre K. Zedaveinyte R. Silanskas A. (2021). Transposon-associated TnpB is a programmable RNA-guided DNA endonuclease. Nature 599, 692–696. 10.1038/s41586-021-04058-1 34619744 PMC8612924

[B30] Kim H. K. Min S. Song M. Jung S. Choi J. W. Kim Y. (2018). Deep learning improves prediction of CRISPR–Cpf1 guide RNA activity. Nat. Biotechnol. 36, 239–241. 10.1038/nbt.4061 29431740

[B31] Kim N. Kim H. K. Lee S. Seo J. H. Choi J. W. Park J. (2020). Prediction of the sequence-specific cleavage activity of Cas9 variants. Nat. Biotechnol. 38, 1328–1336. 10.1038/s41587-020-0537-9 32514125

[B32] Kleine B. Chattopadhyay A. Polen T. Pinto D. Mascher T. Bott M. (2017). The three-component system EsrISR regulates a cell envelope stress response in *Corynebacterium glutamicum* . Mol. Microbiol. 106, 719–741. 10.1111/mmi.13839 28922502

[B33] Kleinstiver B. P. Sousa A. A. Walton R. T. Tak Y. E. Hsu J. Y. Clement K. (2019). Engineered CRISPR–Cas12a variants with increased activities and improved targeting ranges for gene, epigenetic and base editing. Nat. Biotechnol. 37, 276–282. 10.1038/s41587-018-0011-0 30742127 PMC6401248

[B34] Kudla G. Murray A. W. Tollervey D. Plotkin J. B. (2009). Coding-sequence determinants of gene expression in *Escherichia coli* . Science 324, 255–258. 10.1126/science.1170160 19359587 PMC3902468

[B35] Li M. Chen J. Wang Y. Liu J. Huang J. Chen N. (2020). Efficient multiplex gene repression by CRISPR-dCpf1 in *Corynebacterium glutamicum* . Front. Bioeng. Biotechnol. 8, 357. 10.3389/fbioe.2020.00357 32391351 PMC7193084

[B36] Liu J. Liu M. Shi T. Sun G. Gao N. Zhao X. (2022). CRISPR-assisted rational flux-tuning and arrayed CRISPRi screening of an L-proline exporter for L-proline hyperproduction. Nat. Commun. 13, 891–906. 10.1038/s41467-022-28501-7 35173152 PMC8850433

[B37] Liu J. Srinivasan S. Li C. Y. Ho I. L. Rose J. Shaheen M. (2019). Pooled library screening with multiplexed Cpf1 library. Nat. Commun. 10, 3144. 10.1038/s41467-019-10963-x 31316073 PMC6637147

[B38] Liu J. Wang Y. Lu Y. Zheng P. Sun J. Ma Y. (2017). Development of a CRISPR/Cas9 genome editing toolbox for *Corynebacterium glutamicum* . Microb. Cell Fact. 16, 205. 10.1186/s12934-017-0815-5 29145843 PMC5693361

[B39] Liu Y D. L. Dong J. Chen C. Zhu J. Rao V. B. Tao P. (2020). Covalent modifications of the bacteriophage genome confer a degree of resistance to bacterial CRISPR systems. J. Virol. 94, e01630. 10.1128/jvi.01630-20 PMC7654273 32938767

[B40] Magnusson J. P. Rios A. R. Wu L. Qi L. S. (2021). Enhanced Cas12a multi-gene regulation using a CRISPR array separator. Elife 10, e66406. 10.7554/elife.66406 34499031 PMC8478413

[B41] Moreb E. A. Hutmacher M. Lynch M. D. (2020). CRISPR-cas "Non-Target" sites inhibit on-target cutting rates. CRISPR J. 3, 550–561. 10.1089/crispr.2020.0065 33346713

[B42] Moreb E. A. Lynch M. D. (2021). Genome dependent Cas9/gRNA search time underlies sequence dependent gRNA activity. Nat. Commun. 12, 5034. 10.1038/s41467-021-25339-3 34413309 PMC8377084

[B43] Najm F. J. Strand C. Donovan K. F. Hegde M. Sanson K. R. Vaimberg E. W. (2017). Orthologous CRISPR–Cas9 enzymes for combinatorial genetic screens. Nat. Biotechnol. 36, 179–189. 10.1038/nbt.4048 29251726 PMC5800952

[B44] Okafor I. C. Singh D. Wang Y. Jung M. Wang H. Mallon J. (2019). Single molecule analysis of effects of non-canonical guide RNAs and specificity-enhancing mutations on Cas9-induced DNA unwinding. Nucleic Acids Res. 47, 11880–11888. 10.1093/nar/gkz1058 31713616 PMC7145707

[B45] Ozcan A. Krajeski R. Ioannidi E. Lee B. Gardner A. Makarova K. S. (2021). Programmable RNA targeting with the single-protein CRISPR effector Cas7-11. Nature 597, 720–725. 10.1038/s41586-021-03886-5 34489594

[B46] Pan X. Tang M. You J. Osire T. Sun C. Fu W. (2022). PsrA is a novel regulator contributes to antibiotic synthesis, bacterial virulence, cell motility and extracellular polysaccharides production in *Serratia marcescens* . Nucleic Acids Res. 50, 127–148. 10.1093/nar/gkab1186 34893884 PMC8754645

[B47] Park J. Lim J. M. Jung I. Heo S. J. Park J. Chang Y. (2021). Recording of elapsed time and temporal information about biological events using Cas9. Cell 184, 1047–1063. 10.1016/j.cell.2021.01.014 33539780

[B48] Park J B. S. Kim J. S. (2015). Cas-Designer: a web-based tool for choice of CRISPR-Cas9 target sites. Bioinformatics 31, 4014–4016. 10.1093/bioinformatics/btv537 26358729

[B49] Pátek M. Nešvera J. (2013). Corynebacterium glutamicum Biology and Biotechnology. Berlin, Germany: Springer, 51–88.

[B50] Pattanayak V. Lin S. Guilinger J. P. Ma E. Doudna J. A. Liu D. R. (2013). High-throughput profiling of off-target DNA cleavage reveals RNA-programmed Cas9 nuclease specificity. Nat. Biotechnol. 31, 839–843. 10.1038/nbt.2673 23934178 PMC3782611

[B51] Pausch P. Al-Shayeb B. Bisom-Rapp E. Tsuchida C. A. Li Z. Cress B. F. (2020). CRISPR-CasΦ from huge phages is a hypercompact genome editor. Science 369, 333–337. 10.1126/science.abb1400 32675376 PMC8207990

[B52] Peng F. Wang X. Sun Y. Dong G. Yang Y. Liu X. (2017). Efficient gene editing in *Corynebacterium glutamicum* using the CRISPR/Cas9 system. Microb. Cell Fact. 16, 201. 10.1186/s12934-017-0814-6 29137643 PMC5686833

[B53] Perez-Arellano I. Rubio V. Cervera J. (2006). Mapping active site residues in glutamate-5-kinase. The substrate glutamate and the feed-back inhibitor proline bind at overlapping sites. FEBS Lett. 580, 6247–6253. 10.1016/j.febslet.2006.10.031 17069808

[B54] Resende B. C. Rebelato A. D'Afonseca V. Santos A. Stutzman T. Azevedo V. (2011). DNA repair in Corynebacterium model. Gene 482, 1–7. 10.1016/j.gene.2011.03.008 21497183

[B55] Riesenberg S. Helmbrecht N. Kanis P. Maricic T. Paabo S. (2022). Improved gRNA secondary structures allow editing of target sites resistant to CRISPR-Cas9 cleavage. Nat. Commun. 13, 489. 10.1038/s41467-022-28137-7 35078986 PMC8789806

[B56] Singh D. Wang Y. Mallon J. Yang O. Fei J. Poddar A. (2018). Mechanisms of improved specificity of engineered Cas9s revealed by single-molecule FRET analysis. Nat. Struct. Mol. Biol. 25, 347–354. 10.1038/s41594-018-0051-7 29622787 PMC6195204

[B57] Sternberg S. H. LaFrance B. Kaplan M. Doudna J. A. (2015). Conformational control of DNA target cleavage by CRISPR-Cas9. Nature 527, 110–113. 10.1038/nature15544 26524520 PMC4859810

[B58] Sternberg S. H. Redding S. Jinek M. Greene E. C. Doudna J. A. (2014). DNA interrogation by the CRISPR RNA-guided endonuclease Cas9. Nature 507, 62–67. 10.1038/nature13011 24476820 PMC4106473

[B59] Talas A. Huszár K. Kulcsár P. I. Varga J. K. Varga É. Tóth E. (2021). A method for characterizing Cas9 variants via a one-million target sequence library of self-targeting sgRNAs. Nucleic Acids Res. 49, e31. 10.1093/nar/gkaa1220 33450024 PMC8034649

[B60] Tao P. Wu X. Tang W. C. Zhu J. Rao V. (2017). Engineering of bacteriophage T4 genome using CRISPR-cas9. ACS Synth. Biol. 6, 1952–1961. 10.1021/acssynbio.7b00179 28657724 PMC5771229

[B61] Tsuchida C. A. Zhang S. Doost M. S. Zhao Y. Wang J. O’Brien E. (2022). Chimeric CRISPR-CasX enzymes and guide RNAs for improved genome editing activity. Mol. Cell 82, 1199–1209. 10.1016/j.molcel.2022.02.002 35219382 PMC9189900

[B62] Vlot M. Houkes J. Lochs S. J. Swarts D. C. Zheng P. Kunne T. (2018). Bacteriophage DNA glucosylation impairs target DNA binding by type I and II but not by type V CRISPR-Cas effector complexes. Nucleic Acids Res. 46, 873–885. 10.1093/nar/gkx1264 29253268 PMC5778469

[B63] Walton Rt C. K. Whittaker M. N. Kleinstiver B. P. (2020). Unconstrained genome targeting with near-PAMless engineered CRISPR-Cas9 variants. Science 368, 290–296. 10.1126/science.aba8853 32217751 PMC7297043

[B64] Wang B. Hu Q. Zhang Y. Shi R. Chai X. Liu Z. (2018). A RecET-assisted CRISPR–Cas9 genome editing in *Corynebacterium glutamicum* . Microb. Cell Fact. 17, 63. 10.1186/s12934-018-0910-2 29685154 PMC5913818

[B65] Wang D. Zhang C. Wang B. Li B. Wang Q. Liu D. (2019). Optimized CRISPR guide RNA design for two high-fidelity Cas9 variants by deep learning. Nat. Commun. 10, 4284. 10.1038/s41467-019-12281-8 31537810 PMC6753114

[B66] Wang M. Chen B. Fang Y. Tan T. (2017). Cofactor engineering for more efficient production of chemicals and biofuels. Biotechnol. Adv. 35, 1032–1039. 10.1016/j.biotechadv.2017.09.008 28939499

[B67] Wang Y. Cheng H. Liu Y. Liu Y. Wen X. Zhang K. (2021). *In-situ* generation of large numbers of genetic combinations for metabolic reprogramming via CRISPR-guided base editing. Nat. Commun. 12, 678. 10.1038/s41467-021-21003-y 33514753 PMC7846839

[B68] Wang Y. Y. Shi K. Chen P. Zhang F. Xu J. Z. Zhang W. G. (2020). Rational modification of the carbon metabolism of *Corynebacterium glutamicum* to enhance L-leucine production. J. Ind. Microbiol. Biotechnol. 47, 485–495. 10.1007/s10295-020-02282-8 32535763

[B69] Wendisch V. F. (2014). Microbial production of amino acids and derived chemicals: synthetic biology approaches to strain development. Curr. Opin. Biotechnol. 30, 51–58. 10.1016/j.copbio.2014.05.004 24922334

[B70] Wienert B. Cromer M. K. (2022). CRISPR nuclease off-target activity and mitigation strategies. Front. Genome Ed. 4, 1050507. 10.3389/fgeed.2022.1050507 36439866 PMC9685173

[B71] Wolf S. Becker J. Tsuge Y. Kawaguchi H. Kondo A. Marienhagen J. (2021). Advances in metabolic engineering of *Corynebacterium glutamicum* to produce high-value active ingredients for food, feed, human health, and well-being. Essays Biochem. 65, 197–212. 10.1042/ebc20200134 34096577 PMC8313993

[B72] Wu Z. Zhang Y. Yu H. Pan D. Wang Y. Wang Y. (2021). Programmed genome editing by a miniature CRISPR-Cas12f nuclease. Nat. Chem. Biol. 17, 1132–1138. 10.1038/s41589-021-00868-6 34475565

[B73] Xiang X. Corsi G. I. Anthon C. Qu K. Pan X. Liang X. (2021). Enhancing CRISPR-Cas9 gRNA efficiency prediction by data integration and deep learning. Nat. Commun. 12, 3238. 10.1038/s41467-021-23576-0 34050182 PMC8163799

[B74] Xu J X. X. Zhang J. Guo Y. Qian H. Zhang W. (2014). A method for gene amplification and simultaneous deletion in *Corynebacterium glutamicum* genome without any genetic markers. Plasmid 72, 9–17. 10.1016/j.plasmid.2014.02.001 24613758

[B75] Yan M. Y. Yan H. Q. Ren G. X. Zhao J. P. Guo X. P. Sun Y. C. (2017). CRISPR-Cas12a-Assisted recombineering in bacteria. Appl. Environ. Microbiol. 83, e00947–e00917. 10.1128/aem.00947-17 28646112 PMC5561284

[B76] Yarrington R. M. Verma S. Schwartz S. Trautman J. K. Carroll D. (2018). Nucleosomes inhibit target cleavage by CRISPR-Cas9 *in vivo* . Proc. Natl. Acad. Sci. U. S. A. 115, 9351–9358. 10.1073/pnas.1810062115 30201707 PMC6156633

[B77] Yim S. S. An S. J. Kang M. Lee J. Jeong K. J. (2013). Isolation of fully synthetic promoters for high-level gene expression in *Corynebacterium glutamicum* . Biotechnol. Bioeng. 110, 2959–2969. 10.1002/bit.24954 23633298

[B78] Zetsche B. Gootenberg J. Abudayyeh O. Slaymaker I. Makarova K. Essletzbichler P. (2015). Cpf1 is a single RNA-guided endonuclease of a class 2 CRISPR-cas system. Cell 163, 759–771. 10.1016/j.cell.2015.09.038 26422227 PMC4638220

[B79] Zetsche B. Heidenreich M. Mohanraju P. Fedorova I. Kneppers J. DeGennaro E. M. (2017). Multiplex gene editing by CRISPR-Cpf1 using a single crRNA array. Nat. Biotechnol. 35, 31–34. 10.1038/nbt.3737 27918548 PMC5225075

[B80] Zhang J. Qian F. Dong F. Wang Q. Yang J. Jiang Y. (2020). *De novo* engineering of *Corynebacterium glutamicum* for l-proline production. ACS Synth. Biol. 9, 1897–1906. 10.1021/acssynbio.0c00249 32627539

[B81] Zhao D. Badur M. G. Luebeck J. Magaña J. H. Birmingham A. Sasik R. (2018). Combinatorial CRISPR-cas9 metabolic screens reveal critical redox control points dependent on the KEAP1-NRF2 regulatory Axis. Mol. Cell 69, 699–708. 10.1016/j.molcel.2018.01.017 29452643 PMC5819357

[B82] Zhao N. Li L. Luo G. Xie S. Lin Y. Han S. (2020). Multiplex gene editing and large DNA fragment deletion by the CRISPR/Cpf1-RecE/T system in *Corynebacterium glutamicum* . J. Ind. Microbiol. Biotechnol. 47, 599–608. 10.1007/s10295-020-02304-5 32876764

