# Supplementary material for: HyCas9-12aGEP: an efficient genome editing platform for Corynebacterium glutamicum
Source: Front Bioeng Biotechnol. 2024 Mar 12;12:1327172. doi: 10.3389/fbioe.2024.1327172 (PMC10963414; doi:10.3389/fbioe.2024.1327172)
Supplement: Supplementary file 2 [file DataSheet1.PDF]

# Supplementary Information

**HyCas9-12aGEP: An Efficient Genome Editing Platform for *Corynebacterium glutamicum***

**Feng Zhang [a*], Jin-Yu Wang [a], Chang-Lon Li [a], Wei-Guo Zhang [a*]**

# Supplementary methods

**Deletion and insertion in *C. glutamicum* using HyCas9-12aGEP**

1 μg pZF2 series plasmid with a targeting gRNA and two ~1kb HAs was transformed into *C. glutamicum* via electroporation. Transformants were plated on LBH plates supplemented with 25 μg/mL Kan for counter-selection. After 36-48h of cultivation at 30 °C, colonies were verified by PCR.

**Measurement of editing efficiency**

As previously described, this study uses colony PCR to measure the editing efficiency[1]. The data we obtained through colony PCR validation were transformed into editing efficiency. The calculation formula of editing efficiency k is as follows:

$$k=n/m\times 100\%$$

Where $n$ is the number of transformants confirmed as correct by colony PCR; $m$ is the number of transformants randomly selected for colony PCR validation.

**Codon-optimized SpCas9 variant SpRY-HF1 sequence**

The SpCas9 variant SpRY-HF1 contains a mutant combination of the Cas9 variant SpRY-Cas9[2] with near-PAMless requirement and the high-fidelity Cas9 variant SpCas9-HF1[3]. Red bold bases represent the difference between SpRY-Cas9 and SpCas9-encoded amino acid residues. Blue bold bases represent the difference between SpCas9-HF1 and SpCas9-encoded amino acid residues. Codon-optimized SpRY-HF1 gene sequence are available from the NCBI under accession numbers OP345224. The detail sequence is listed as follow:

ATGGATAAAAAGTATTCCATTGGCCTGGACATCGGCACCAATTCTGTGGGTTGGGCAGT
CATCACCGACGAATACAAGGTCCCATCCAAGAAGTTCAAGGTGCTCGGTAATACCGATC
GCCACTCTATCAAGAAAAACCTGATCGGCGCCCTGCTCTTCGACTCCGGCGAAACCGCA
GAA**CGT**ACCCGTCTCAAGCGTACCGCACGTCGCCGCTACACCCGCCGTAAGAATCGCAT
CTGCTACCTCCAGGAAATCTTCTCTAATGAGATGGCAAAGGTGGATGACTCCTTTTTCCA

CCGCCTCGAAGAGTCCTTCCTGGTGGAAGAGGACAAGAAACACGAGCGCCATCCTATCT
TCGGCAATATTGTCGATGAAGTCGCATATCATGAAAAATACCCAACCATTTACCATCTCC
GTAAAAAACTCGTCGATTCCACCGATAAGGCAGATCTCCGCCTGATCTATCTGGCACTG
GCCCACATGATCAAGTTTCGTGGCCACTTCCTGATCGAAGGTGACCTCAATCCAGACAAT
TCCGACGTGGACAAGCTGTTCATCCAGCTGGTGCAAACCTACAACCAGCTCTTTGAGGA
AAACCCAATCAACGCATCTGGCGTCGACGCAAAAGCCATCCTGTCCGCCCGTCTCTCCA
AGTCTCGTCGCCTCGAAAACCTCATTGCCCAGCTCCCTGGCGAGAAGAAAAACGGTCTG
TTCGGCAATCTGATCGCCCTGTCTCTGGGTCTGACCCCAAATTTCAAATCCAACTTTGAT
CTCGCAGAAGATGCCAAGCTGCAGCTCTCTAAGGACACCTACGATGATGACCTGGATAA
CCTCCTCGCCCAGATCGGCGACCAGTACGCCGATCTCTTCCTCGCAGCCAAGAACCTCT
CTGACGCAATTCTGCTGTCCGACATCCTGCGCGTGAACACCGAAATCACTAAGGCACCA
CTCTCTGCCTCCATGATTAAGCGCTACGACGAGCATCATCAGGATCTCACTCTCCTCAAA
GCCCTGGTCCGCCAGCAGCTCCCAGAGAAGTACAAGGAAATCTTTTTCGACCAATCCAA
AAACGGCTACGCAGGTTACATCGATGGCGGCGCCTCTCAGGAAGAGTTTTACAAATTCA
TTAAGCCAATCCTCGAAAAGATGGACGGCACCGAGGAACTGCTGGTGAAACTCAACCGT
GAAGATCTCCTGCGCAAACAGCGCACCTTCGACAACGGTTCCATTCCTCACCAGATCCA
CCTGGGCGAACTGCACGCAATCCTCCGCCGTCAAGAGGACTTCTACCCATTCCTGAAGG
ACAACCGTGAAAAGATCGAAAAGATTCTCACCTTCCGCATCCCTTACTACGTGGGTCCTC
TCGCCCGTGGCAATTCCCGCTTCGCCTGGATGACTCGCAAATCTGAAGAGACCATCACC
CCTTGGAACTTTGAGGAAGTCGTCGATAAAGGCGCCTCTGCCCAATCCTTCATCGAGCG
CATGACC**GCC**TTCGACAAGAACCTCCCAAACGAGAAAGTGCTGCCTAAGCATTCCCTCC
TGTACGAGTACTTTACTGTCTACAATGAGCTGACTAAGGTGAAGTATGTCACTGAAGGCA
TGCGTAAGCCAGCCTTTCTCTCCGGCGAGCAGAAAAAGGCAATCGTCGACCTCCTGTTT
AAAACCAACCGCAAAGTGACTGTGAAACAGCTCAAGGAAGATTACTTTAAAAAAATCGAA
TGCTTCGATTCTGTGGAAATCTCCGGCGTGGAGGATCGTTTCAACGCCTCCCTGGGTAC
CTATCACGACCTCCTGAAGATTATCAAGGACAAAGATTTTCTGGATAACGAGGAAAACGA
GGACATTCTCGAGGACATTGTGCTGACCCTGACCCTCTTCGAGGACCGCGAGATGATCG
AGGAGCGCCTGAAGACCTATGCCCACCTCTTTGACGACAAGGTCATGAAGCAACTCAAG
CGCCGCCGCTATACCGGTTGGGGC**GCC**CTCTCCCGTAAGCTCATCAATGGTATCCGCG
ACAAGCAATCCGGCAAGACTATCCTGGACTTTCTGAAGTCTGACGGCTTCGCCAACCGC
AATTTTATG**GCC**CTGATCCACGACGATTCCCTGACCTTCAAAGAGGACATCCAGAAAGCC
CAAGTGTCCGGTCAAGGCGACTCCCTGCACGAACACATCGCCAATCTGGCAGGTTCCCC
AGCAATCAAGAAGGGCATCCTGCAGACCGTCAAGGTGGTGGACGAACTCGTCAAAGTGA
TGGGTCGCCACAAACCAGAAAACATCGTCATCGAGATGGCCCGTGAGAACCAGACCACC
CAGAAAGGCCAGAAAAACTCCCGTGAGCGCATGAAGCGCATTGAAGAAGGCATTAAAGA
GCTCGGCTCTCAGATCCTGAAAGAGCATCCTGTCGAGAACACCCAACTGCAGAATGAGA
AGCTGTATCTGTATTATCTCCAGAACGGCCGCGACATGTACGTCGACCAGGAACTGGAC
ATCAACCGTCTCTCTGATTACGATGTGGACCATATCGTCCCTCAGTCTTTCCTGAAAGAC
GACTCTATTGACAACAAAGTCCTCACCCGCTCCGACAAGAACCGCGGCAAGTCCGATAA
CGTGCCATCCGAGGAGGTCGTGAAGAAGATGAAGAACTACTGGCGCCAGCTGCTCAAC
GCCAAGCTGATCACTCAGCGCAAGTTCGATAACCTGACCAAGGCCGAACGTGGTGGTCT
CTCCGAGCTCGACAAGGCAGGCTTTATCAAGCGCCAACTCGTGGAGACTCGC**GCC**ATCA
CTAAACACGTCGCCCAGATCCTCGACTCCCGCATGAATACCAAGTACGATGAAAATGAC
AAGCTCATCCGCGAAGTGAAAGTCATTACCCTGAAGTCCAAACTGGTCTCTGACTTTCGC

AAGGATTTCCAGTTCTACAAGGTCCGCGAGATTAATAACTATCATCATGCACACGATGCATACCTCAACGCAGTCGTGGGTACTGCACTGATCAAGAAGTACCCTAAACTGGAGTCCGAGTTCGTCTATGGCGACTACAAGGTGTACGACGTCCGCAAAATGATTGCCAAGTCCGAGCAGGAGATCGGCAAAGCAACTGCCAAATATTTCTTTTACTCCAACATCATGAACTTCTTCAAGACCGAAATCACCCTCGCCAACGGTGAAATCCGCAAACGTCCACTCATCGAGACTAATGGTGAAACCGGCGAGATCGTCTGGGACAAGGGCCGTGACTTCGCAACCGTCCGCAAGGTCCTCTCCATGCCACAGGTCAACATCGTGAAAAAGACCGAGGTGCAAACCGGCGGCTTCTCCAAGGAGTCTATC**CGC**CCTAAACGCAATTCCGATAAGCTCATTGCACGCAAAAAGGACTGGGACCCTAAAAAATACGGCGGTTTC**CTGTGG**CCAACTGTCGCATATTCTGTGCTCGTGGTCGCCAAAGTGGAAAAGGGCAAATCCAAAAAGCTCAAGTCCGTCAAGGAACTCCTGGGTATCACCATCATGGAACGCTCCTCCTTTGAGAAGAACCCTATCGATTTCCTCGAAGCAAAAGGTTACAAGGAGGTGAAGAAAGATCTGATCATCAAGCTCCCTAAATACTCCCTCTTCGAGCTCGAGAACGGCCGCAAGCGTATGCTGGCCTCCGCA**AAGCAA**CTGCAAAAAGGTAACGAGCTGGCACTCCCATCCAAGTATGTCAACTTTCTCTACCTGGCCTCCCACTACGAAAAGCTGAAAGGTTCCCCAGAAGACAACGAGCAGAAACAGCTGTTCGTGGAGCAGCACAAGCACTACCTGGACGAAATCATCGAGCAGATCTCCGAGTTCTCTAAACGCGTCATTCTGGCCGATGCCAACCTCGATAAAGTGCTCTCCGCCTACAATAAGCATCGTGATAAGCCAATCCGTGAGCAGGCAGAGAACATCATTCACCTGTTCACTCTCACC**CGT**CTGGGTGCACCA**CGT**GCCTTTAAGTACTTCGACACCACCATCGAC**CCA**AAG**CAG**TAT**CGC**TCCACTAAGGAGGTGCTCGATGCAACCCTGATCCACCAGTCTATCACCGGCCTCTACGAGACTCGCATCGATCTCTCCCAGCTGGGTGGCGACTAA

# Supplementary Figures

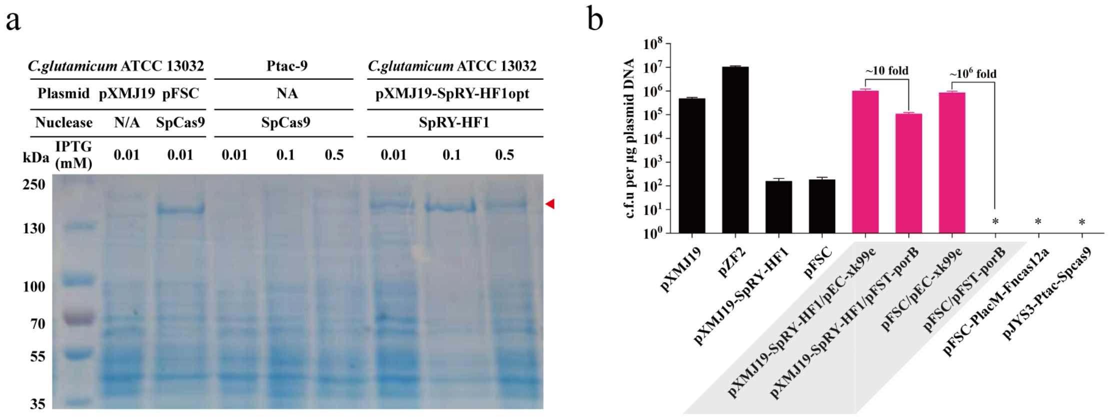


**Figure S1. Expression test for SpRY-HF1 and SpCas9 in *C. glutamicum*. (a)** Expression of spCas9 (~160 kDa) and SPY-HF1 from *C. glutamicum* ATCC 13032 or Ptac-9. Ptac-9 and *C. glutamicum* ATCC 13032 harboring plasmid pFSC (pXMJ19-spCas9opt) or pXMJ19-SpRY-HF1opt were cultured in LBG medium for testing spCas9 and SpRY-HF1 expression. IPTG was supplemented at different concentrations (0.01 – 0.5 mM). Red arrows (►) indicate overexpressed protein bands. **(b)** Transformation efficiency of different plasmids electroporated into *C. glutamicum* ATCC13032. The gray shaded part represents the co-transformation of *C. glutamicum* 13032 with the two-plasmid system for testing the counter-selection activity of nucleases SpCas9 and SpRY-HF1. For the two-plasmid system, IPTG was added at 10 µM. * means no transformant.

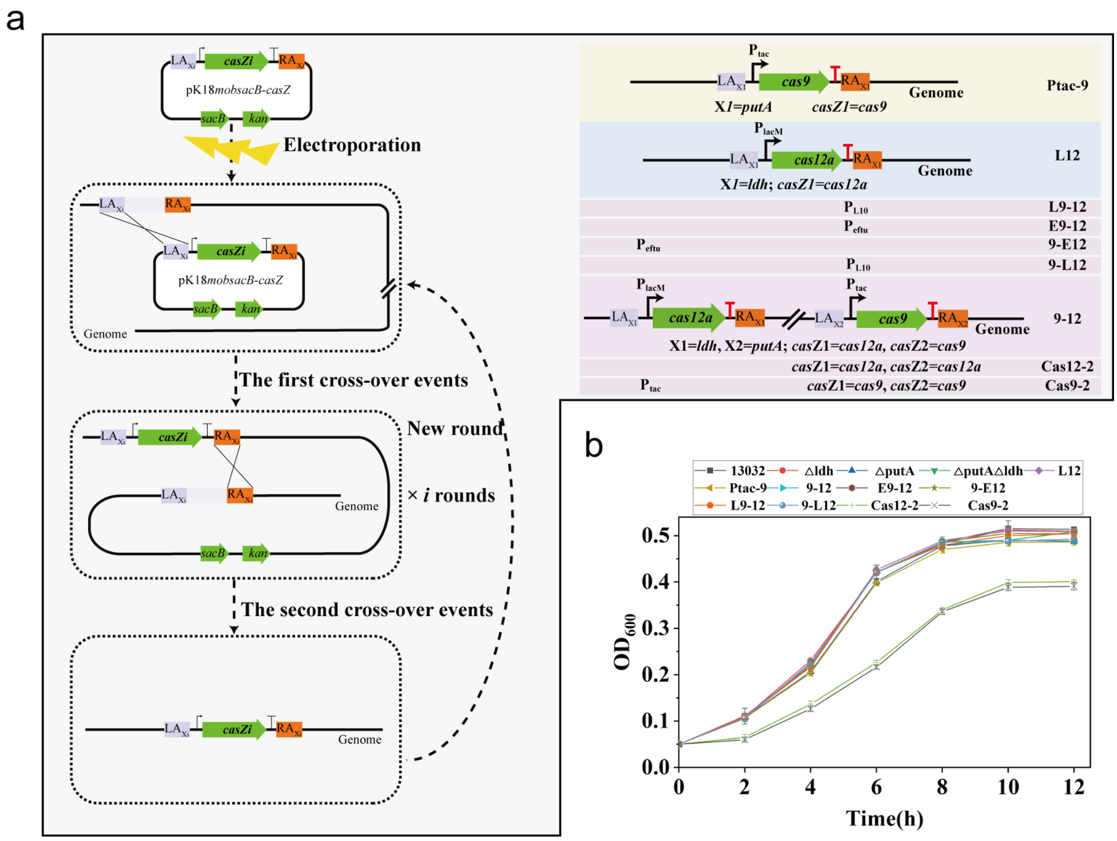


**Figure S2. Construction of genome-integrated strains with different *cas* genes and determination of growth curves. (a)** Schematic of the integration of *cas9* or *cas12a* into the *C. glutamicum* genome. *casZ* represents genes encoding Cas9 or Cas12a and dCas9. ***i*** represents the number of iterative integrations, and *casZ**i*** represents the *casZ* gene integrated in the ***i*** round. Xi represents the site of integration of the *cas* gene. *casZ* represents the integrated *cas* gene. Ptac-9: *C. glutamicum* ATCC 13032△*putA::*$P_{tac}$*-cas9-rrnBT1T2*; L12: *C. glutamicum* ATCC 13032△*ldh::*$P_{lacM}$*-cas12a-rrnBT1T2*; L9-12: *C. glutamicum* ATCC 13032△*putA::*$P_{L10}$*-cas9-rrnBT1T2*△*ldh::*$P_{lacM}$*-cas12a-rrnBT1T2*; 9-E12: *C. glutamicum* ATCC 13032△*putA::*$P_{tac}$*-cas9-rrnBT1T2*△*ldh::*$P_{eftu}$*-cas12a-rrnBT1T2*; E9-12: *C. glutamicum* ATCC 13032△*putA::*$P_{eftu}$*-cas9-rrnBT1T2*△*ldh::*$P_{lacM}$*-cas12a-rrnBT1T2*; 9-12: *C. glutamicum* ATCC 13032△*putA::*$P_{tac}$*-cas9-rrnBT1T2*△*ldh::*$P_{lacM}$*-cas12a-rrnBT1T2*; 9-L12: *C. glutamicum* ATCC 13032△*putA::*$P_{tac}$*-cas9-rrnBT1T2*△*ldh::*$P_{L10}$*-cas12a-rrnBT1T2*; Cas12-2: *C. glutamicum* ATCC 13032△*putA::*$P_{lacM}$*-cas9-rrnBT1T2*△*ldh::*$P_{lacM}$*-cas12a-rrnBT1T2*; Cas9-2: *C. glutamicum* ATCC 13032△*putA::*$P_{tac}$*-cas9-rrnBT1T2*△*ldh::*$P_{tac}$*-cas9-rrnBT1T2*; **(b)** Growth curves of strains carrying *cas* genes in different ways. The $OD_{600}$ value is the measurement result of a 25-fold dilution. △*ldh*: *C. glutamicum* ATCC13032 strain with knocked out of *ldh*; △*putA*: *C. glutamicum*

ATCC13032 strain with knocked out of *putA*; △*ldh*△*putA* refers to the *C. glutamicum* ATCC13032 strain with knocked out of *ldh* and *putA* genes.

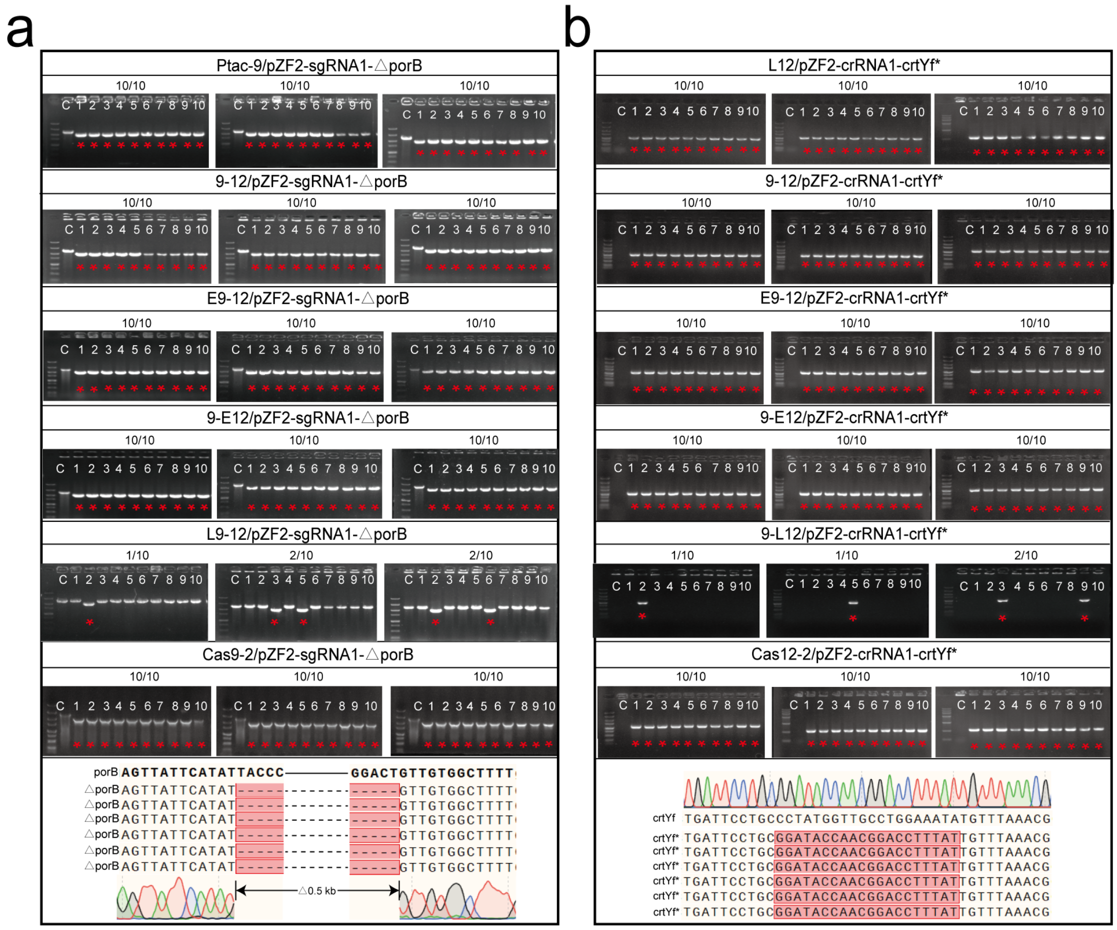


**Figure S3. PCR validation of colonies obtained by crRNA1 and sgRNA1-mediated gene editing experiments.** Colonies derived from genome editing experiments, as indicated in Figure 1b, c, f and g, were screened by colony PCR, and the positive recombinants were further identified by sequencing. *(n/N): n, number of editing-positive transformants; N, number of transformants tested. For *porB*(0.5kb) deletion, positive recombinants of 2.0kb are indicated in agarose gel, while the wild genotype are 2.5kb. For the MAMA PCR[4, 5] verification of point mutation *crtYf*, no band for wild-type control was obtained, because the verification primer could not bind to the genomic DNA of wild-type but only to the DNA of mutant recombinant. Thus, no band is indicative of the wild-type genotype, whereas the presence of 1.0 kb fragments is indicative of recombinant genotypes. DNA ladder (DL 5000 DNA Marker, Vazyme Biotech Co.,Ltd) was used as a marker. **C**: *C. glutamicum* ATCC13032 genomic DNA was used as negative template controls. Three independent replicates were conducted.

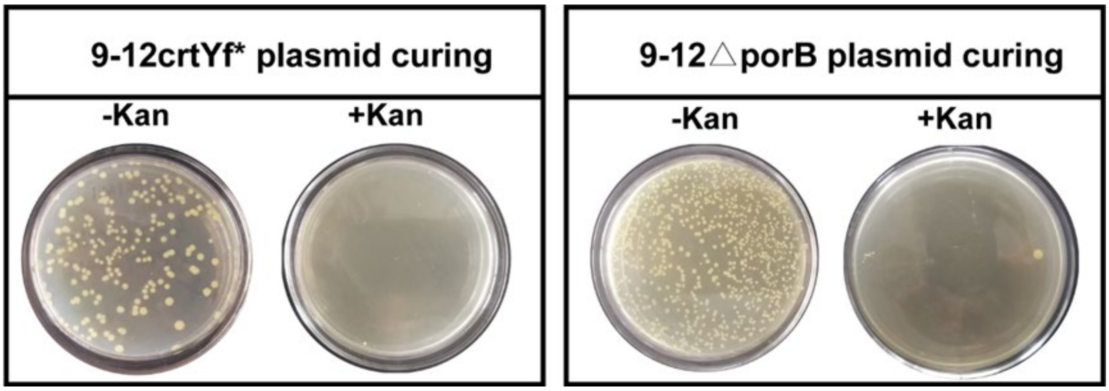


**Figure S4. Evaluation of the plasmid curing efficiency of the temperature-sensitive plasmid pZF2.** The single colonies of 9-12crtYf*/pZF2-crRNA1-crtYf* and 9-12△porB/pZF2-sgRNA1-△porB were inoculated into 50 mL shake-flask containing 10 mL of LBG medium and incubated overnight at 37°C with agitation. Subsequently, they were diluted and evenly spread onto chloramphenicol-resistant and chloramphenicol-sensitive CM agar plates, followed by quantification of transformants.

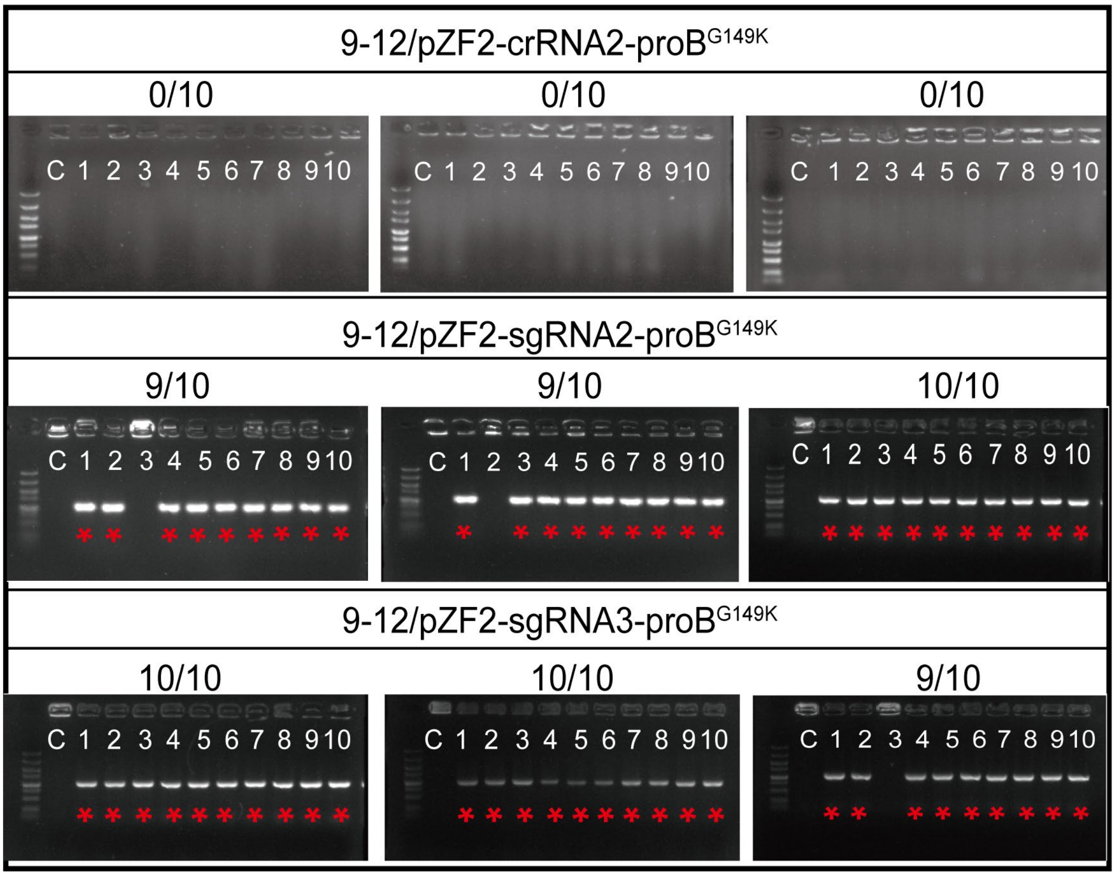


**Figure S5. The colony PCR validation results for gene editing of the ProB$^{G149K}$ point mutation mediated by crRNA2, sgRNA2 and sgRNA3.** Colonies derived from point mutation *proB* experiment, as indicated in **Figure 2c**, were randomly selected for colony PCR verification. For the MAMA PCR[4, 5] verification of point mutation *proB*, no band for wild-type control was obtained, because the verification primer could not bind to the genomic DNA of wild-type but only to the DNA of mutant recombinant. Thus, no band is indicative of the wild-type genotype, whereas the presence of 1.05 kb fragments is indicative of recombinant genotypes. Correctly edited strains are marked with red stars in agarose gel. DNA ladder (DL 5000 DNA Marker, Vazyme Biotech Co.,Ltd) was used as a marker. C: *C. glutamicum* ATCC13032 genomic DNA was used as negative template controls. Three independent replicates were conducted.

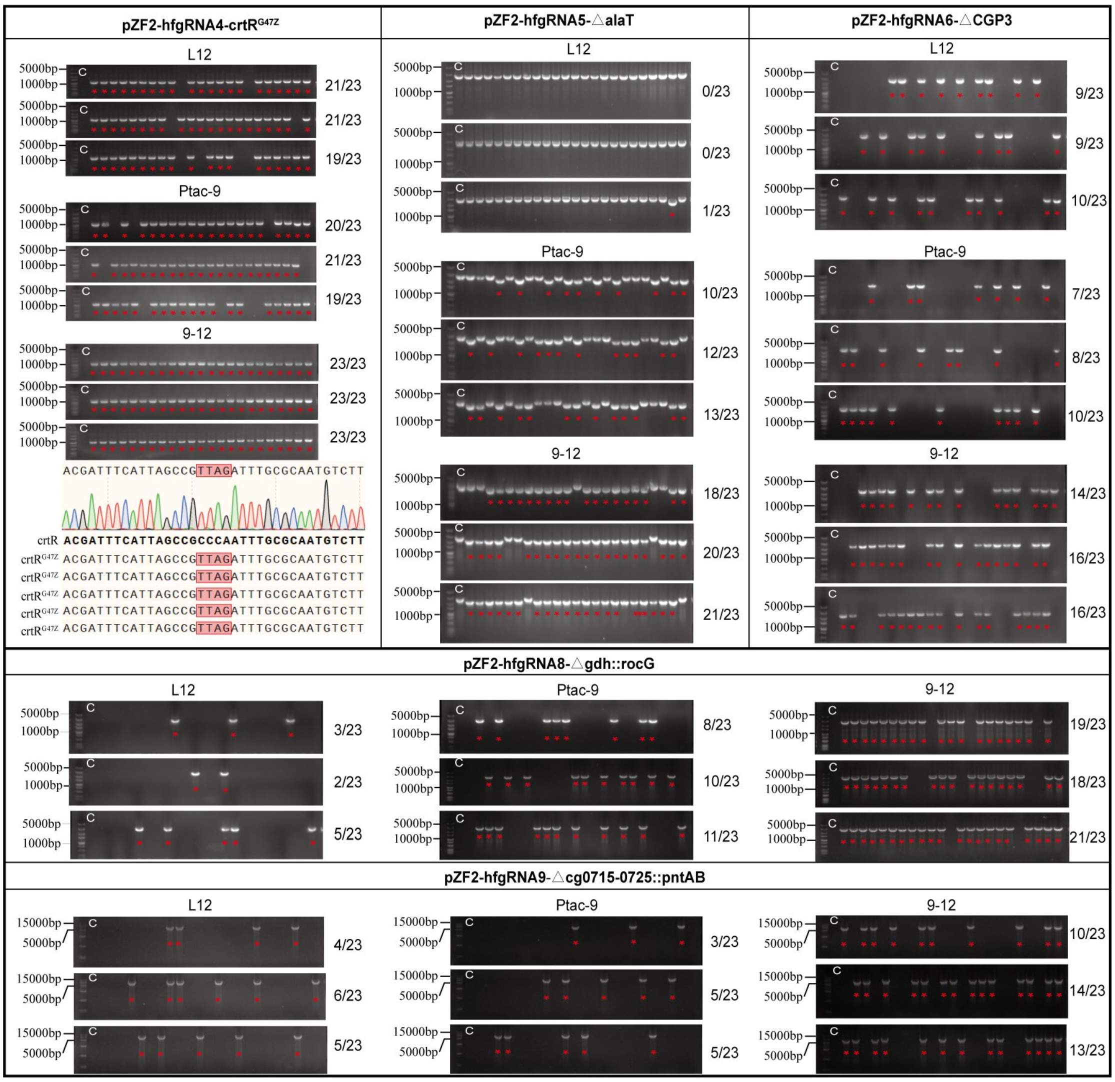


**Figure S6. PCR validation of colonies obtained by hfgRNA4-8-mediated gene editing experiments.** Colonies derived from genome editing experiments, as indicated in Figure 4c, d, e, f, g and Figure S6, were screened by colony PCR. *(n/N): n, number of editing-positive transformants; N, number of transformants tested. For the MAMA PCR[4, 5] verification of point mutation CrtR$^{G47Z}$, no band is indicative of the wild-type genotype, whereas the presence of 1.05 kb fragments is indicative of recombinant genotypes. The positive CrtR$^{G47Z}$ recombinants were further identified by sequencing. For *alaT* gene deletion (0.5kb), positive recombinants of 2.0 kb are marked in agarose gel, while the wild genotype are 2514 bp. For CGP3(219kb) deletion and H36-*pntAB-rrnBT1T2*(3.5kb) insertion, no band was obtained for the wild genotype because the target >12 kb fragment was too large for PCR experiment conducted in this test, while positive recombinants of 2.5 kb and 5.5kb are indicated in agarose gel, respectively. For the insertion of P*tac-rocG-rrnBT1T2*, one PCR validation primer is located within the foreign

inserted fragment, while the other is positioned outside the homologous arms within the genome. Consequently, only transformants with successful insertion will yield effective PCR amplification. no band was obtained for the wild genotype, while positive recombinants of 2.7kb are marked in agarose gel. Correctly edited strains are marked with red stars in agarose gel. DNA ladder (DL 5000 and 15000 DNA Marker, Vazyme Biotech Co.,Ltd) was used as a marker. C: *C. glutamicum* ATCC13032 genomic DNA was used as negative template controls. Three independent replicates were conducted.

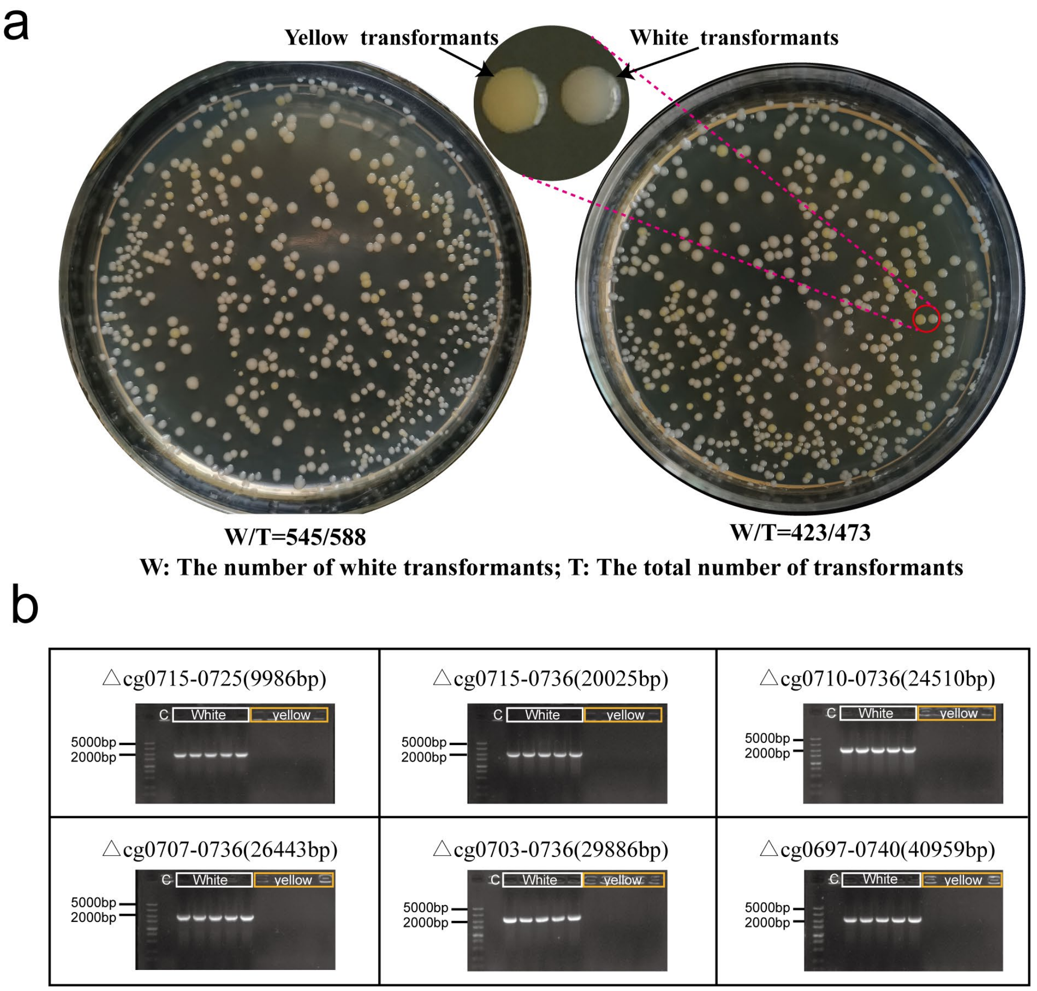


**Figure S7. Colony color variation and colony PCR validation results for large segment gene deletion mediated by hfgRNA9.** (**a**) Colony morphology of transformants for cg0715-0725 gene deletion mediated by hfgRNA9 on LBHIS agar plates. For deletions targeting *cg0715-0725*, *cg0715-0736*, *cg0710-0736*, *cg0707-0736*, *cg0703-0736* and *cg0697-0740*, transformants on the plates exhibited both white and yellow colonies. Here, we are presenting the color variations of transformants for the cg0715-0725 deletion. (**b**) PCR validation of five randomly selected white and yellow colonies from LBHIS agar plates. For *cg0715-0725*, *cg0715-0736*, *cg0710-0736*, *cg0707-0736*, *cg0703-0736* and *cg0697-0740* deletion, no band was obtained for the wild genotype because the target >12 kb fragment was too large for PCR experiment conducted in this test, while positive recombinants of 2.0 kb, 2.0kb, 2.0kb, 2.0kb, 2.2kb and 2.1kb are indicated in agarose gel, respectively. Correctly edited strains are marked with red stars in agarose gel. In LBHIS agar plates, white transformants all exhibited correct

editing, while yellow strains were identified as escape cells. DNA ladder (DL 5000 and 15000 DNA Marker, Vazyme Biotech Co.,Ltd) was used as a marker. C: *C. glutamicum* ATCC13032 genomic DNA was used as negative template controls. Three independent replicates were conducted.

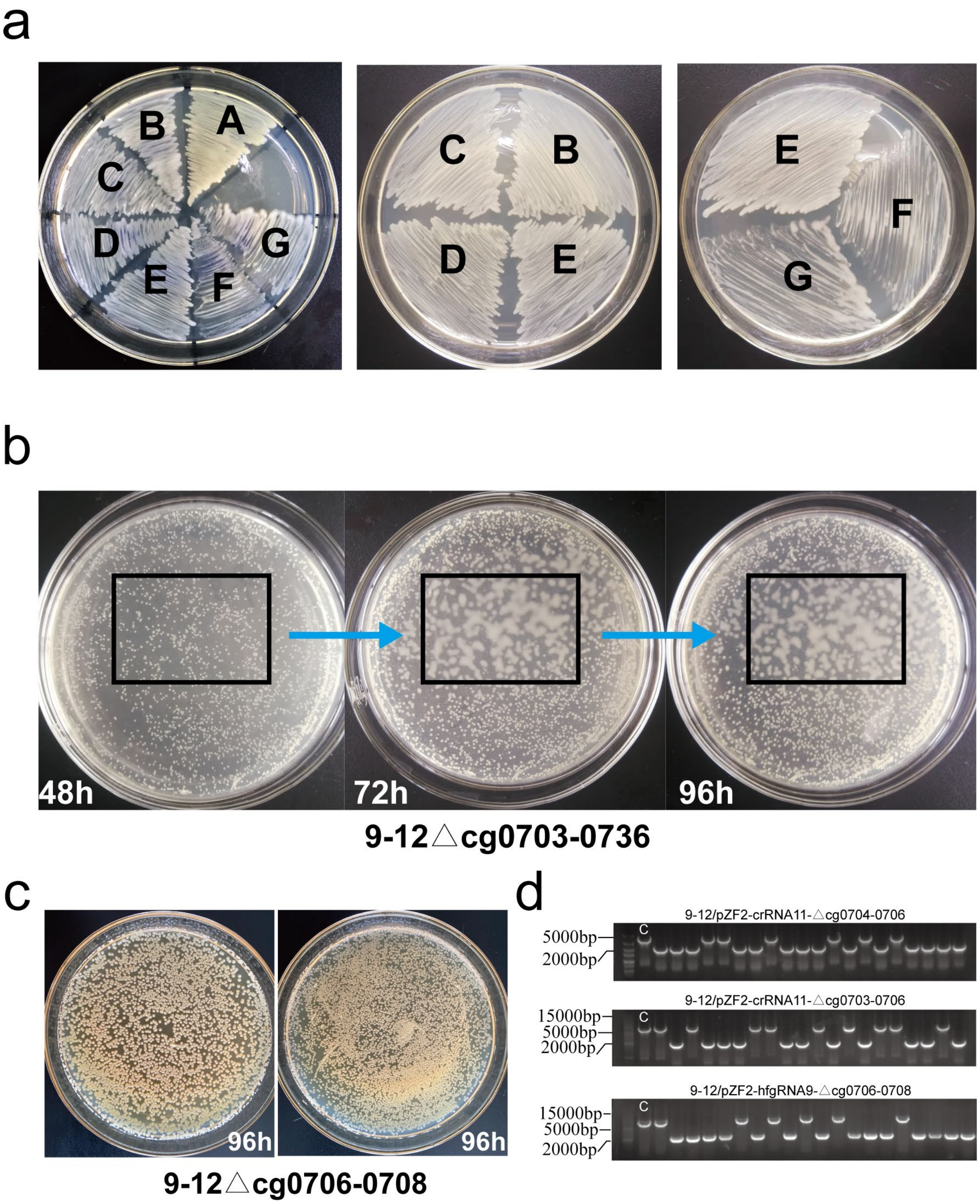


**Figure S8. The influence of deletions in different gene fragments on colony phenotypes.** **(a)** The effects of deleting different gene fragments on colony color and growth variations. A:9-12; B:9-12△cg0715-0725 (9.986kb); C:9-12△cg0715-0725 (20.02kb); D:9-12△cg0710-0725 (24.51kb); E:9-12△cg0707-0725 (26.44kb); F:9-12△cg0703-0736 (29.86kb); G:9-12△cg0697-0740 (40.96 kb). **(b)** Colony morphological changes in 9-12△cg0703-0736 strain at 48h, 72h, and 96h. Use black boxes to highlight changes in colony morphology. **(c)** Colony morphological characteristics of the 9-12△cg0703-0736 strain cultured at 30℃ for 96h. **(d)** The

gene deletion colony PCR validation was conducted using crRNA11 and hfgRNA9. crRNA11 mediated the deletion of cg0704-0706 (3.58kb) and cg0704-0706 (1.95kb), while hfgRNA9 mediated the deletion of cg0706-0708 (3.20kb). DNA ladder (DL 5000 and 15000 DNA Marker, Vazyme Biotech Co.,Ltd) was used as a marker. C: *C. glutamicum* ATCC13032 genomic DNA was used as negative template controls.

# Supplementary Tables

## Table S1. List of the strain in this study

| Strain | Characteristics | Source/Ref. |
|---|---|---|
| *E. coli* JM109 | recA1, supE44 endA1 hsdR17 (r−k,m+ k) gyrA96 relA1 thi (lac-proAB) F'[traD36 proAB+ lacIq lacZΔM15] | Lab stock |
| *C. glutamicum* ATCC 13032 | Type strain | Lab stock |
| *C. glutamicum* S9114 | Glutamate-producing strain | Lab stock |
| *C. glutamicum* LG-3 | Glutamine-producing strain derived from *C. glutamicum* ATCC 13870. | Lab stock |
| *C. glutamicum* ATCC 13869 | Type strain | Lab stock |
| *C. glutamicum* N-77 | Lysine-producing strain derived from *C. glutamicum* ATCC 13761. | Lab stock |
| *C. glutamicum* I31-5 | Lysine-producing strain derived from *C. glutamicum* ATCC 17965. | Lab stock |
| △*putA*△*ldh* | *C. glutamicum* ATCC 13032△*putA*△*ldh* | This study |
| Ptac-9 | *C. glutamicum* ATCC 13032△*putA*::$P_{tac}$-*cas9* | This study |
| L12 | *C. glutamicum* ATCC 13032△*ldh*::$P_{lacM}$-*cas12a* | This study |
| 9-12 | *C. glutamicum* ATCC 13032△*putA*::$P_{tac}$-*cas9*△*ldh*::$P_{lacM}$-*cas12a* | This study |
| E9-12 | *C. glutamicum* ATCC 13032△*ldh*::$P_{lacM}$-*cas12a*△*putA*::$P_{eftu}$-*cas9* | This study |
| L9-12 | *C. glutamicum* ATCC 13032△*ldh*::$P_{lacM}$-*cas12a*△*putA*::$P_{L10}$-*cas9* | This study |
| 9-E12 | *C. glutamicum* ATCC 13032△*putA*::*Ptac-cas9*△*ldh*::$P_{eftu}$-*cas12a* | This study |
| 9-L12 | *C. glutamicum* ATCC 13032△*putA*::*Ptac-cas9*△*ldh*::$P_{L10}$-*cas12a* | This study |
| Cas9-2 | *C. glutamicum* ATCC 13032△*putA*::$P_{tac}$-*cas9*△*ldh*::$P_{tac}$-*cas9* | This study |
| Cas12a-2 | *C. glutamicum* ATCC 13032△*putA*:: $P_{lacM}$-*cas12a*△*ldh*::$P_{lacM}$-*cas12a* | This study |
| 9-12(S9114) | *C. glutamicum* S9114△*putA*::$P_{tac}$-*cas9*△*cg0716*::$P_{lacM}$-*cas12a* | This study |
| 9-12(LG-3) | *C. glutamicum* LG-3△*putA*::$P_{tac}$-*cas9*△*cg0716*::$P_{lacM}$-*cas12a* | This study |
| 9-12(ATCC 13869) | *C. glutamicum* ATCC 13869△*putA*::$P_{tac}$-*cas9*△*cg0716*::$P_{lacM}$-*cas12a* | This study |
| 9-12(N-77) | *C. glutamicum* N-77△*putA*::$P_{tac}$-*cas9*△*cg0716*::$P_{lacM}$-*cas12a* | This study |
| 9-12(I31-5) | *C. glutamicum* I31-5△*putA*::$P_{tac}$-*cas9*△*cg0716*::$P_{lacM}$-*cas12a* | This study |
| 9-12proB$^{G149K}$ | *C. glutamicum* ATCC 13032△*putA*::$P_{tac}$-*cas9*△*ldh*::$P_{lacM}$-*cas12a proB*$^{G149K}$ | This study |
| 9-12crtYf$^{*}$ | *C. glutamicum* ATCC 13032△*putA*::$P_{tac}$-*cas9*△*ldh*::$P_{lacM}$-*cas12a crtYf*$^{*}$ | This study |
| 9-12△porB | *C. glutamicum* ATCC 13032△*putA*::$P_{tac}$-*cas9*△*ldh*::$P_{lacM}$-*cas12a*△*porB* | This study |
| 9-12crtR$^{G47Z}$ | *C. glutamicum* ATCC 13032△*putA*::$P_{tac}$-*cas9*△*ldh*::$P_{lacM}$-*cas12a crtR*$^{G47Z}$ | This study |
| 9-12△alaT | *C. glutamicum* ATCC 13032△*putA*::$P_{tac}$-*cas9*△*ldh*::$P_{lacM}$-*cas12a*△alaT | This study |
| 9-12△CGP3 | *C. glutamicum* ATCC 13032△*putA*::$P_{tac}$-*cas9*△*ldh*::$P_{lacM}$-*cas12a*△CGP3 | This study |
| 9-12△gdh::rocG | *C. glutamicum* ATCC 13032△*putA*::$P_{tac}$-*cas9*△*ldh*::$P_{lacM}$-*cas12a*△gdh::Ptac-rocG | This study |
| 9-12△0715-0725::pntAB | *C. glutamicum* ATCC 13032△*putA*::$P_{tac}$-*cas9*△*ldh*::$P_{lacM}$-*cas12a*△*0715-0725*::$P_{H36}$-*pntAB* | This study |
| 9-12△*g0715-0725*(9986bp) | *C. glutamicum* ATCC 13032△*putA*::$P_{tac}$-*cas9*△*ldh*::$P_{lacM}$-*cas12a*△*cg0715-0725* | This study |
| 9-12△*cg0715-0736*(20025bp) | *C. glutamicum* ATCC 13032△*putA*::$P_{tac}$-*cas9*△*ldh*::$P_{lacM}$-*cas12a*△*cg0715-0736* | This study |
| 9-12△*cg0710-0736*(24510bp) | *C. glutamicum* ATCC 13032△*putA*::$P_{tac}$-*cas9*△*ldh*::$P_{lacM}$-*cas12a*△*cg0710-0736* | This study |
| 9-12△*cg0707-* | *C. glutamicum* ATCC 13032△*putA*::$P_{tac}$-*cas9*△*ldh*::$P_{lacM}$-*cas12a*△*cg0707-* | This study |

| | | |
|---|---|---|
| *0736*(26443bp) | *0736* | |
| 9-12△*cg0703-0736*(29868bp) | *C. glutamicum* ATCC 13032△*putA*::$P_{tac}$-*cas9*△*ldh*::$P_{lacM}$-*cas12a*△*cg0703-0736* | This study |
| 9-12△*cg0697-0740*(40959bp) | *C. glutamicum* ATCC 13032△*putA*::$P_{tac}$-*cas9*△*ldh*::$P_{lacM}$-*cas12a*△*cg0697-0740* | This study |
| 9-12△*cg0704-cg0706* | *C. glutamicum* ATCC 13032△*putA*::$P_{tac}$-*cas9*△*ldh*::$P_{lacM}$-*cas12a*△*cg0704-cg0706* | This study |
| 9-12△*cg0703-cg0706* | *C. glutamicum* ATCC 13032△*putA*::$P_{tac}$-*cas9*△*ldh*::$P_{lacM}$-*cas12a*△*cg0703-cg0706* | This study |

**Table S2. Purchased and donated plasmids in this study.**

| Plasmid | **Source/Ref.** |
|---|---|
| pFSC | [1] |
| pFST-porB | [1] |
| pXM-gfp | [6] |
| pJYS3 | Addgene |
| pJYS2_crtYf | Addgene |

## Table S3. Results of the large DNA fragment deletion in 9-12, Ptac-9 and L12

| Size of DNA fragments deleted (bp) | Host cell | Results. (W/T) | Efficiency (%) |
|---|---|---|---|
| 9986 | 9-12 | (545/588), (423/473) | 92.68, 89.43 |
| | L12 | (349/602), (406/654) | 57.97, 62.07 |
| | Ptac-9 | (83/356), (41/207) | 23.31, 19.80 |
| 20025 | 9-12 | (123/178), (99/134) | 69.10, 73.88 |
| | L12 | (54/259), (39/155) | 20.84, 25.16 |
| | Ptac-9 | (1/26), (2/17) | 3.84, 11.76 |
| 24510 | 9-12 | (27/50), (22/35) | 54.00, 62.85 |
| | L12 | / | / |
| | Ptac-9 | / | / |
| 26443 | 9-12 | (10/18), (13/22) | 59.1, 55.6 |
| | L12 | / | / |
| | Ptac-9 | / | / |
| 29868 | 9-12 | (8/18), (8/16) | 44.4, 50.0 |
| | L12 | / | / |
| | Ptac-9 | / | / |
| 40959 | 9-12 | (4/11), (4/10) | 36.3, 40.0 |
| | L12 | (0/16), (0/33) | 0, 0 |
| | Ptac-9 | (0/2), (0/9) | 0, 0 |

W, the number of white transformants; T, the total number of transformants; /, untested; Efficiency, probability of deletion events occurring, calculated as W/T ×100%.

**Table S4.** Off target analysis of edited strains.

| | 9-12 | 9-12△*porB* | 9-12△*cg0697-0740* | 9-12△*gdh::rocG* |
|---|---|---|---|---|
| Genome size (bp) | 3,287,270 | 3,286,771 | 3,246,311 | 3,287,507 |
| Hiseq date (Mb) | 352 | 350 | 348 | 350 |
| CDS SNP | 0 | 0 | 0 | 0 |
| Intergenic SNP | 0 | 0 | 0 | 0 |
| CDS Indel | 2 | 3 | 42 | 3 |
| Intergenic Indel | 2 | 2 | 2 | 3 |

Summary of variant type found in 9-12, 9-12△*porB*, 9-12△*cg0697-0740* and 9-12△*gdh::rocG* strain. Genome size is the genome size of each sample; Hiseq date is the date size of High-throughput sequencing for each sample; CDS SNP is the number of SNP occurred in CDS regions; Intergenic SNP is the number of SNP generated in intergenic region; CDS Indel and Intergenic Indel are the number of Indel generated in CDS regions and Intergenic region, respectively.

# References


1. Peng, F. et al. Efficient gene editing in *Corynebacterium glutamicum* using the CRISPR/Cas9 system. *Microb. Cell Fact.* **16**, 201 (2017).
2. Walton RT, C.K., Whittaker MN, Kleinstiver BP. Unconstrained genome targeting with near-PAMless engineered CRISPR-Cas9 variants. *Science* **368**, 290-296 (2020).
3. Chen, J.S. et al. Enhanced proofreading governs CRISPR-Cas9 targeting accuracy. *Nature* **550**, 407-410 (2017).
4. Kwok S, K.D., McKinney N, Spasic D, Goda L, Levenson C, Sninsky JJ. Effects of primer-template mismatches on the polymerase chain reaction: human immunodeficiency virus type 1 model studies. *Nucleic Acids Res.* **18(4):999-1005** (1990 ).
5. Deekshit, V.K. et al. Mismatch amplification mutation assay-polymerase chain reaction: A method of detecting fluoroquinolone resistance mechanism in bacterial pathogens. *Indian J Med Res* **149**, 146-150 (2019).
6. Li, M. et al. Efficient Multiplex Gene Repression by CRISPR-dCpf1 in *Corynebacterium glutamicum*. *Front. Bioeng. Biotechnol.* **8**, 357 (2020).
